# Precipitation Dominates Forest Net Primary Productivity Variations With Distinct Regional Differences in Yunnan Province, China

**DOI:** 10.1002/ece3.72893

**Published:** 2026-01-04

**Authors:** Xiaofang Yang, Kun Yang, Shaohua Zhang, Wenxia Zeng, Jing Liu, Yan Rao, Yan Ma, Changyou Bi

**Affiliations:** ^1^ Faculty of Geography Yunnan Normal University Kunming China; ^2^ GIS Technology Research Center of Resource and Environment in Western China, Ministry of Education Yunnan Normal University Kunming China; ^3^ Southwest United Graduate School Kunming China

**Keywords:** forest NPP, multi‐scale analysis, nonlinear threshold responses, stable forest and changing forest

## Abstract

In the topographically and climatically diverse region of Yunnan, clarifying the driving mechanisms and threshold effects of factors influencing forest net primary productivity (NPP) is crucial for managing forest carbon sinks. In this study, we established a comparative analysis framework for the stable forest (SF) and the changing forest (CF). Yunnan was then divided into five subregions based on topography and climate. Using a random forest model and the SHAP method, we systematically analyzed drivers of spatiotemporal NPP variations. The results show that CF exhibits greater NPP variation and spatial heterogeneity than SF and maintains stronger trend persistence. At the provincial scale, elevation and precipitation are the main drivers of NPP in SF and CF, respectively, while at the subregional scale, dominant factors differ and include solar radiation, temperature, and forest age, indicating clear scale‐dependent effects. The explanatory power of multiple‐factor interactions for NPP variations is generally higher than that of single factors, with precipitation showing particularly strong synergistic effects. Human activities also have a pronounced impact on NPP in CF. Key driving factors exhibit nonlinear threshold responses, with NPP in SF most suitable under temperature (14°C–20°C), precipitation (100–125 mm), elevation (~2000 m), and forest age (50–70 years), whereas CF shows broader response ranges, reflecting greater adaptability. This study highlights the nonlinear responses and threshold characteristics of forest NPP to multiple driving factors in complex mountainous environments. It also emphasizes the importance of interactions and scale effects in ecological modeling.

## Graphical abstract

We first classified the forests in Yunnan Province into stable forest and change forest regions, and then further divided the province into five sub‐regions based on topography and climate. Based on this classification, we systematically analyzed the spatiotemporal variation of forest net primary productivity (NPP) across these regions. Moreover, by employing the Random Forest model and SHAP analysis, we identified the key driving factors behind the NPP changes in different sub‐regions.

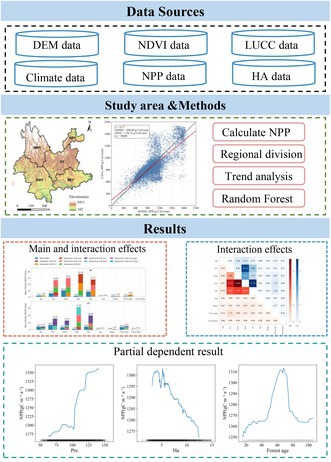

## 1 Introduction

Against the backdrop of accelerating global climate change, forests are the largest carbon sink in terrestrial ecosystems and play a central role in achieving dual‐carbon targets through their carbon sequestration capacity (Felipe‐Lucia et al. 2018; Ma et al. 2025; Pan et al. 2011). Net Primary Productivity (NPP) is a key indicator of the carbon sink strength of forest ecosystems and reflects the efficiency of vegetation in fixing carbon through photosynthesis (Wei et al. 2022). Therefore, NPP is of fundamental importance in research on the carbon cycle.

However, forest NPP is shaped by multiple factors, including topography, climate, and human activities. In mountainous regions with complex terrain and diverse climates, its spatiotemporal heterogeneity becomes particularly evident (Li et al. 2021; Liu et al. 2019). At the macro scale, precipitation and temperature are often considered the dominant controls. Yet at the regional scale, especially in complex ecosystems that experience strong human disturbance, the relative importance of different drivers, their interactions, and the nonlinear or threshold effects they impose on NPP remain insufficiently understood. This cognitive gap arises mainly from two sources. First, spatial differences in natural geographical conditions and in the intensity of human disturbance lead to distinct NPP response mechanisms across regions. For example, temperature and light may be the main limiting factors in humid areas (Karnieli et al. 2019; Liu, Zohner, et al. 2025), while precipitation becomes critical in arid and semi‐arid regions (Zhang et al. 2023). At the same time, human activities such as urban expansion (Mu et al. 2023) and agricultural reclamation (Forzieri et al. 2022) can reduce NPP, whereas ecological restoration projects (Huang et al. 2020; Wang et al. 2017) may support its recovery. Second, most existing studies emphasize large‐scale analyses that often treat forests as homogeneous units (Gong et al. 2023; Prăvălie et al. 2023; Xi et al. 2023). Such an approach does not effectively distinguish the response mechanisms of primary forests from those of disturbed forest areas, and it lacks systematic exploration of nonlinear interaction mechanisms within regions.

More importantly, the response of forest NPP to driving factors often exhibits nonlinearity and threshold effects (Hong et al. 2024; Li and Zhang 2025). When environmental stress exceeds the critical tolerance of an ecosystem, it may trigger abrupt changes in system structure and function (Zhao et al. 2024). However, research on NPP threshold behavior remains limited, and traditional statistical methods and models have constraints in revealing such complex mechanisms. For example, meta‐analyses (Hillebrand et al. 2020) tend to overlook the heterogeneity of ecological data, conventional regressions (Seddon et al. 2016) struggle to capture nonlinear relationships and interactions among variables, and system dynamics models (Sheldrick et al. 2016) have limited capacity in handling complex dynamic processes. These factors collectively restrict a deeper understanding of the complex response mechanisms of NPP.

In recent years, machine learning methods have provided a new approach to address these challenges. The random forest model has become an important tool in ecological research due to its efficiency in capturing nonlinear relationships and its resistance to overfitting (Wu et al. 2022). Combined with the SHAP interpretability algorithm (Jin et al. 2025), it can clearly quantify the contribution and interaction effects of each factor on the prediction outcomes, and it has been successfully applied in fields such as urban ecology (Deng et al. 2025) and carbon emissions (Li, Sun, et al. 2025).

To address the limitations of current studies in analyzing regional heterogeneity and nonlinear mechanisms, this research takes Yunnan Province, China, as a representative case. Yunnan spans a complete vertical vegetation spectrum from tropical zones to alpine regions and exhibits pronounced climatic gradients (Cui et al. 2022), making it an ideal model system for investigating complex mountain ecosystems. Most existing studies examine forest NPP at the provincial scale and focus on its responses to climate and topography (Chen and Zhang 2023; He et al. 2022), yet they have not fully revealed the divergent characteristics among different ecological subsystems. To address this gap, we first divided Yunnan into five geographic subregions based on its topographic and climatic features to capture the spatial heterogeneity of NPP. We then introduced a comparative framework of the stable forest and the changing forest to distinguish the effects of intrinsic ecological processes from those driven by external disturbances on carbon sink functions. Within this refined framework, defined by the intersection of geographic subregions and dynamic forest types, we combined random forest models with SHAP interpreters to systematically investigate the drivers of forest NPP. The specific objectives of this study are: (1) to clarify the spatial patterns and temporal trends of NPP across subregions, (2) to identify and quantify the independent contributions and interaction effects of climatic, topographic, and human activity factors on NPP, and (3) to reveal the nonlinear influences and potential threshold behaviors of key driving factors.

This study uses the complex mountain forests of Yunnan Province as a case. It combines a geographic zoning approach with a stable‐change dual perspective to analyze the nonlinear effects of driving factors on NPP. The results help to understand regional differences in forest carbon sink function and provide guidance for ecological management and climate change mitigation. In addition, the approach and empirical findings can serve as a reference for forest carbon cycle studies in other complex terrain regions worldwide.

## 2 Data and Methods

### 2.1 Study Area

Yunnan Province is located in southwestern China (97°31′–106°11′, 21°8′–29°15′), covering a total area of about 3.941 × 10^5^ km^2^. The province is dominated by mountainous plateaus, with high terrain in the northwest and low terrain in the southeast, and significant differences in elevation, forming a diverse climate zone ranging from tropical rainforests to alpine frigid zones. The average annual precipitation is about 1066 mm, and the average annual temperature is about 15.8°C. Benefiting from its unique topography and climatic conditions, Yunnan Province has rich forest resources with diverse vegetation types, with a forest stock of 1.973 billion cubic meters and a forest coverage rate of 55.04% (Tu et al. 2023). At the end of 2023, the province's population totaled 46.73 million, GDP exceeded 300 billion yuan, and the impact of human activities on the natural environment was relatively significant.

**FIGURE 1:**
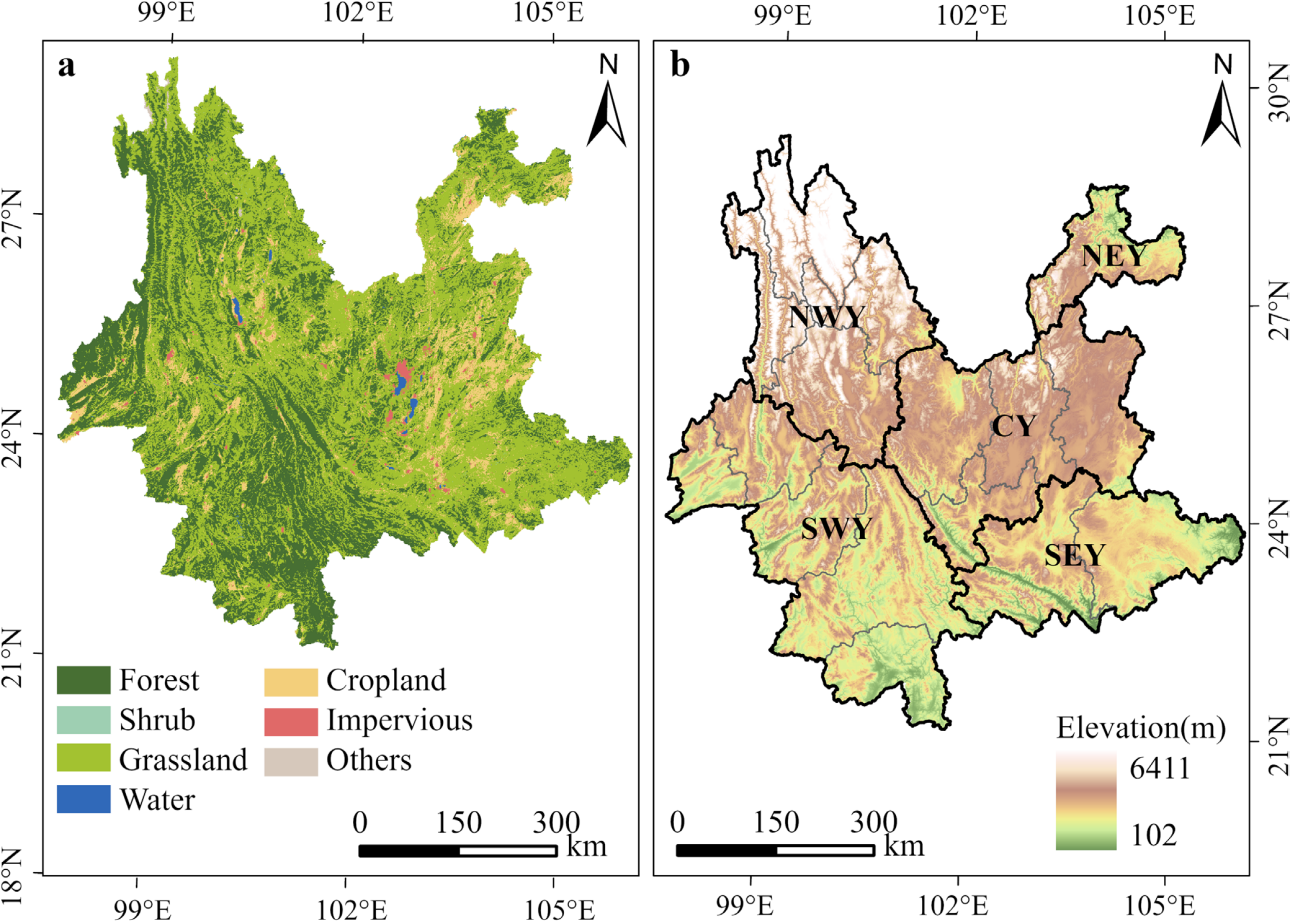
(a) Land use pattern of Yunnan Province in 2020, (b) Distribution of altitude in Yunnan Province.

In the paper, Yunnan Province is divided into five subregions: northeast Yunnan (NEY), central Yunnan (CY), northwest Yunnan (NWY), southwest Yunnan (SWY), and southeast Yunnan (SEY), based on Yan et al. (2021) criteria (Figure 1). Each region has its unique geographic and climatic characteristics.

### 2.2 Data Sources

#### 2.2.1 Basic Data

The land cover type data were obtained from the MCD12Q1 dataset (https://modis.gsfc.nasa.gov/), which has a spatial resolution of 500 m and a temporal resolution of 1 year, spanning from 2001 to 2022. In this paper, the IGBP classification scheme was used, with an accuracy of 73.6% on a global scale. The data have been validated for forest area within the study region using high‐resolution imagery (Table S1). The NPP data are derived from the National Aeronautics and Space Administration (NASA) MOD17A3HGF v061 dataset (https://lpdaac.usgs.gov) for the years 2001 to 2022, with a spatial resolution of 500 m and a temporal resolution of 1a. NDVI data were obtained from the MOD13A1 dataset (https://search.earthdata.nasa.gov/), with a spatial resolution of 500 m and processed using the maximum value composite method on a monthly basis.

#### 2.2.2 Data on Drivers

This study selected driving factors from both natural and anthropogenic dimensions to systematically analyze the mechanisms of driving changes in forest NPP (Table 1). The anthropogenic factor was measured by the anthropogenic intensity data, which integrates eight variables such as built environment, population density, and nighttime lighting to provide a comprehensive picture of the different aspects of human pressure. The precision of this dataset (*R* ^2^ = 0.62) was superior to human footprint maps used in previous studies (Mu et al. 2022). Data on the distribution of plantations and natural forests were obtained from (Xiao et al. 2024), with a classification accuracy of 86% in the Asian region. Forest age data were obtained from (Shang et al. 2025), and validation showed an error of ±2.48 years for disturbed forests and ±7.91 years for undisturbed forests. For ease of use, all data were resampled to 500 m.

**TABLE 1:** Information on the driver data.

Dataset	Unit	Resolution	Data resources
Monthly average precipitation (*Pre*)	mm	1000 m	1‐km monthly precipitation dataset for China (1901–2023) (https://www.tpdc.ac.cn)
Monthly average temperature (*Tem*)	°C	1000 m	1‐km monthly minimum temperature dataset for China (1901–2023) (https://www.tpdc.ac.cn)
Monthly average solar radiation (*Srad*)	W/m^2^	1/24°	https://www.climatologylab.org/terraclimate.html
Slope (*Slope*)	°	90 m	Geospatial Data Cloud (https://www.gscloud.cn)
Elevation (*Ele*)	m	90 m	Geospatial Data Cloud (https://www.gscloud.cn)
Aspect (Asp)	m	90 m	Geospatial Data Cloud (https://www.gscloud.cn)
Human activity intensity (*Ha*)	—	1000 m	A global record of annual terrestrial Human Footprint dataset from 2000 to 2018 (https://doi.org/10.1038/s41597‐022‐01284‐8)
Plantations/Natural forests (forest type)	—	30 m	Global Natural and Planted Forests Mapping at Fine Spatial Resolution of 30 m (https://spj.science.org/doi/10.34133/remotesensing.0204)
Forest age (forest age)	year	30 m	China's annual forest age dataset at a 30 m spatial resolution from 1986 to 2022 (https://essd.copernicus.org/articles/17/3219/2025)

### 2.3 Methods

#### 2.3.1 CASA Model

This study estimated forest NPP using the CASA model, which is based on the principle of light use efficiency (Lv et al. 2024; Zhang, Wang, et al. 2024; Zhu et al. 2007). The core equation of the model is:

(1) $$\mathrm{NPP}\left(x,t\right)=\text{APAR}\left(x,t\right)\times \varepsilon \left(x,t\right)$$

Here, net primary productivity is calculated as the product of the absorbed photosynthetically active radiation (APAR) and the actual light use efficiency (ε). The model derives APAR from remote sensing vegetation indices. It adjusts the potential light use efficiency ($\varepsilon^{*}$) using temperature and moisture stress coefficients to account for environmental limitations on photosynthesis. The detailed parameters of $\varepsilon^{*}$ are provided in Table S2. This approach enables high‐precision simulation of NPP at regional scales.

#### 2.3.2 Regional Division

In this study, forests were classified into two dynamic types using a Geoinformation Tupu method (Wang et al. 2023). The stable forest (SF) maintained the same forest subclass throughout the study period, while the changing forest (CF) included transitions between forest and non‐forest or shifts among forest subclasses. This classification aimed to distinguish the differential impacts of natural fluctuations and human activities on carbon sink function. The classification was performed on the ArcGIS 10.8 platform by spatially overlaying land use data from 2001 to 2022. The formula is presented below (Chen et al. 2020):

(2) C=10A+B

where C represents the changed attribute value, A and B are the land use raster values at the beginning and end of the study period, respectively. A weighting factor of 10 is applied to ensure the uniqueness and clarity of land use type changes. For instance, a value of 12 indicates the conversion from evergreen needleleaf forests to evergreen broadleaf forests. On this basis, we constructed a nested analytical framework. Within the five major natural geographic subregions (Figure 1), we analyzed the NPP variation patterns and driving factors of the stable forest and the changing forest separately.

#### 2.3.3 Trend Analysis

In this study, the Theil–Sen trend analysis method, as well as the Mann–Kendall trend test method, was used to analyze the trend of forest NPP and test the significance. The Theil–Sen trend analysis method (Sen 1968; Theil 1950) is usually used for trend analysis of long time series with the following formula:

(3) $$\rho =\text{Median}\left(\frac{\mathrm{NPP}j-\mathrm{NPP}i}{j-i}\right),2001⩽i<j⩽2022$$

where Median() represents the median of all values. If ρ > 0, the forest NPP shows an increasing trend; if ρ = 0, there is no change; if ρ < 0, the trend is decreasing.

The Mann–Kendall trend test (Kendall 1938; Mann 1945) is a nonparametric test that is often used as a complement to the Theil–Sen trend analysis method of estimation to test for the significance of a time series.

#### 2.3.4 Hurst Index

The Hurst index (*H*) (Zhou et al. 2020) is widely used in the quantitative analysis of the persistence of changes in time series, and is particularly important in the study of the relationship between the annual average NPP and its backward and forward changes. The basic principle is that the past annual average NPP condition has an impact on the current state, and the current state will also have an impact on the future trend. The value of *H* ranges from 0 to 1. When *H* ∈ [0, 0.5), the future trend of annual average NPP is opposite to the current. When *H* = 0.5, the annual average NPP series shows unsustainable stochastic fluctuation. When *H* ∈ (0.5, 1], the future trend of annual average NPP will be consistent with the current. To deeply analyze the future evolution trend of the annual average NPP, this paper combines the Hurst index with the trend analysis method for superposition processing, and the specific calculation method can be referred to the literature (Jiang et al. 2015). The classification criteria are provided in Table S3.

#### 2.3.5 Random Forest Model and SHAP Values

Random Forest (RF) (Breiman 2001), as a nonlinear machine learning method, has been widely used to analyze the relationship between NPP and its drivers in research due to its excellent overfitting resistance and interpretability advantages (Liu et al. 2024). In this study, we applied RF and the Shapley Additive exPlanations (SHAP) interpretive framework based on game‐theoretic Shapley values to systematically explore the response mechanism of forest NPP to driving factors.

RF, as a representative of ensemble learning, effectively captures complex nonlinear associations among variables by constructing multiple decision trees trained on random subsets of data. Its built‐in feature importance assessment automatically identifies the key drivers affecting NPP changes. To ensure optimal generalization ability, a 7:3 ratio is used to divide the training and test sets, and model parameter tuning is performed based on the Bayesian optimization algorithm. The model performance is then evaluated using the coefficient of determination (*R* ^2^) and root mean square error (RMSE).

In order to better reveal the complex interactions among driving factors and enhance model interpretability, this study introduces the SHAP analysis method (Zhang, Chang, and Liu 2024). SHAP quantifies the contribution of each feature to individual predictions, illustrating clearly the relationship between features and NPP changes. In addition, SHAP analyzes the interaction effects among features and identifies higher‐order interactions among variables. Additionally, this study uses partial dependence plots (PDPs) (Grange and Carslaw 2019) generated by the RF model to visualize the threshold effect of a single feature on the predicted value of the NPP and further deepen the understanding of the NPP driving mechanism. The specific calculation method is provided in (Hong et al. 2025).

## 3 Analysis of Results

### 3.1 Accuracy Assessment of NPP Estimated by the CASA Model

**FIGURE 2:**
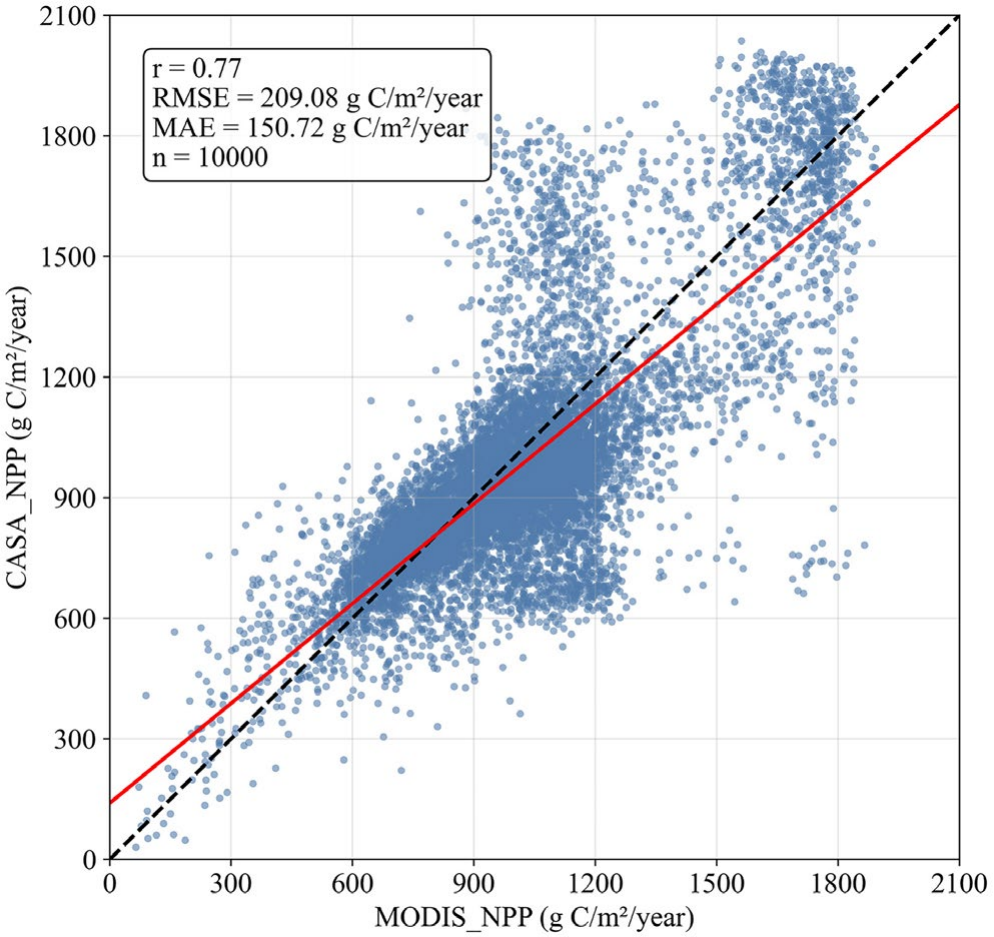
Accuracy validation of NPP simulations using the CASA model.

To verify the reliability and accuracy of the NPP simulation results, this study selected the MOD17A3 product as reference data for consistency checking. The results showed a coefficient of determination (*R* ^2^) of 0.77 (Figure 2), indicating that the NPP results obtained are highly reliable and meet the requirements for subsequent analysis.

### 3.2 Spatial and Temporal Distribution Characteristics of Forest NPP

**FIGURE 3:**
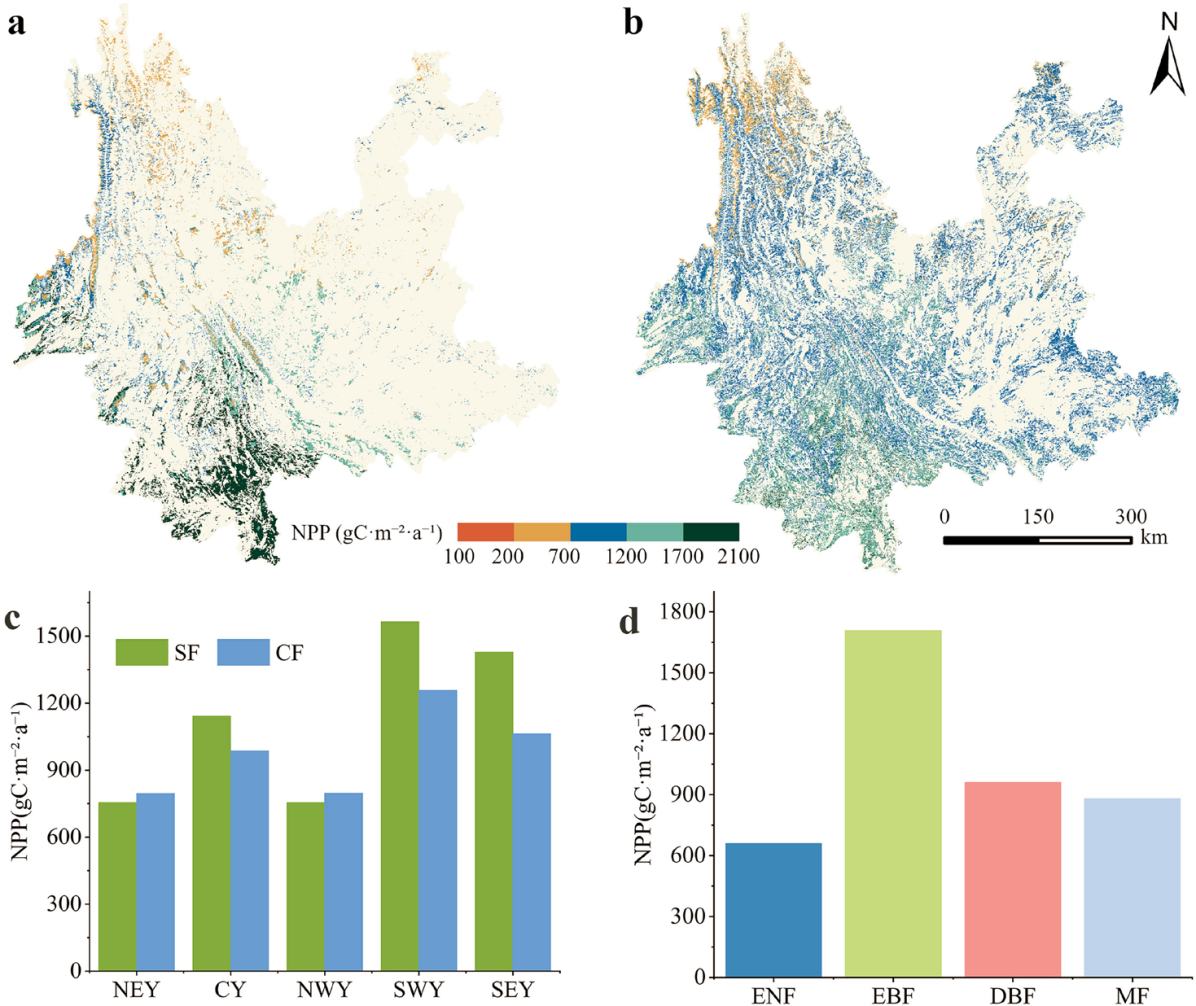
(a) Spatial distribution of the multi‐year mean NPP in the stable forest (SF), (b) spatial distribution of the multi‐year mean NPP in the changing forest (CF), (c) multi‐year mean NPP across all regions, and (d) multi‐year mean NPP of each forest type within the stable forest.

Forest NPP in Yunnan Province shows a spatial pattern of high values in the south and west and low values in the north and east (Figure 3). The long‐term mean NPP of the stable forest is 1334.94 gC·m^−2^·a^−1^, with the highest value in southwestern Yunnan (1564.61 gC·m^−2^·a^−1^), followed by southeastern Yunnan (1427.78 gC·m^−2^·a^−1^). Central Yunnan shows lower values (1141.79 gC·m^−2^·a^−1^), while northeastern and northwestern Yunnan are similar at about 755 gC·m^−2^·a^−1^, reflecting a clear latitudinal decline. The mean annual NPP of the changing forest is 1050.03 gC·m^−2^·a^−1^, overall lower than that of the stable forest (Figure 3c).

Significant differences are observed among forest types (Figure 3d). Evergreen broadleaf forests (EBF) show the highest NPP at 1707.59 gC·m^−2^·a^−1^, which is substantially higher than that of deciduous broadleaf forests (DBF, 960.45 gC·m^−2^·a^−1^), mixed forests (MF, 880.16 gC·m^−2^·a^−1^), and evergreen needleleaf forests (ENF, 658.91 gC·m^−2^·a^−1^).

### 3.3 Changing Characteristics of Forest NPP

**FIGURE 4:**
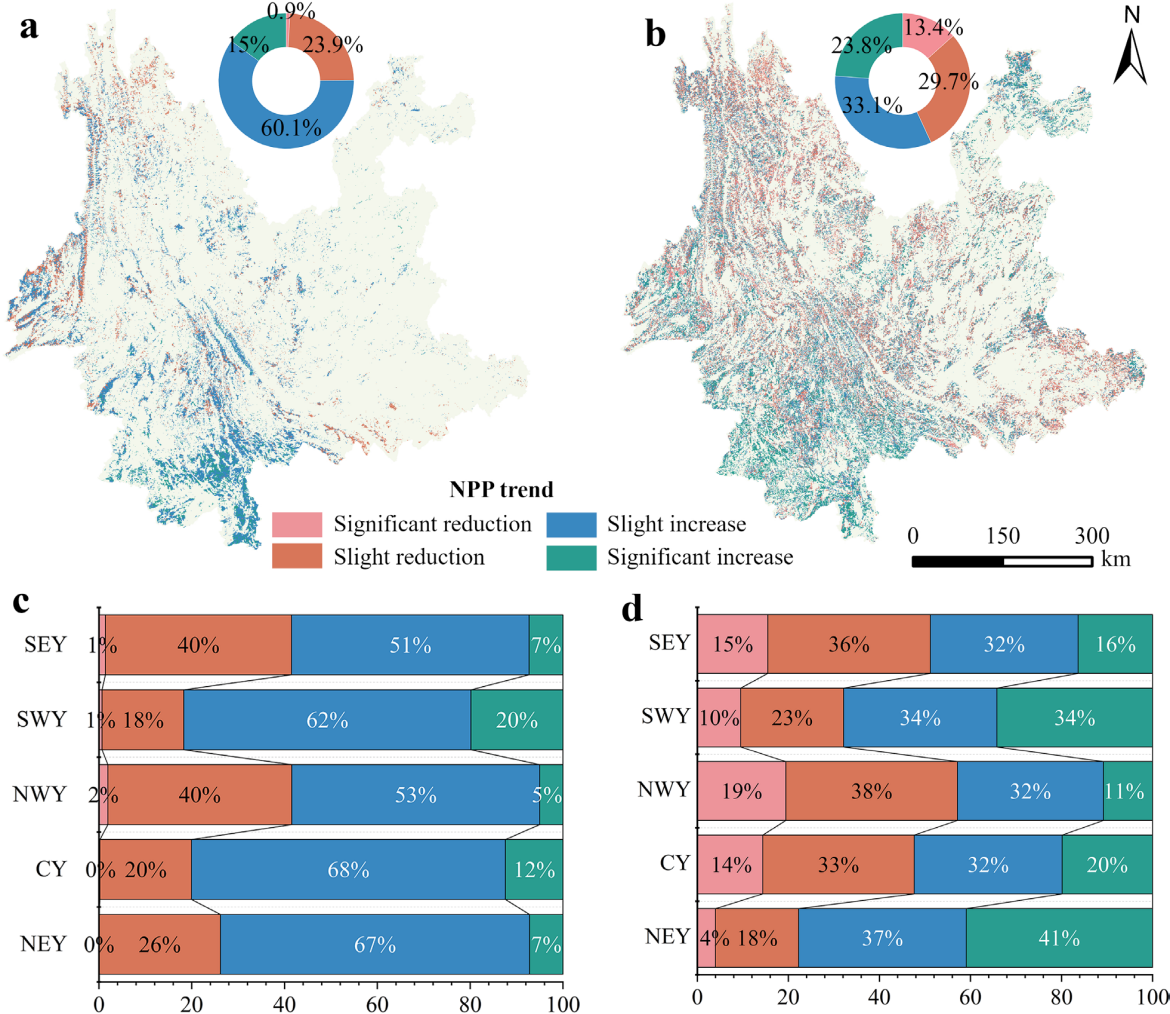
(a) Spatial distribution and area proportion of NPP change trends in the stable forest, (b) spatial distribution and area proportion of NPP change trends in the changing forest, (c) area proportion of NPP change trends in the stable forest subregions, and (d) area proportion of NPP change trends in the changing forest subregions.

In Yunnan Province, the NPP trend of the stable forest shows an overall pattern of improvement (Figure 4). Approximately 75.1% of the stable forest area exhibits an increasing NPP trend, with 15% showing a significant increase, mainly in Xishuangbanna and Pu'er. Slight increases account for 60.1% and are widely distributed across most parts of the province. NPP decreases occur in 24.8% of the stable forest area, of which slight decreases account for 23.9% and are mainly located in the high‐altitude regions of northwestern Yunnan. Areas with significant decreases account for less than 1% (Figure 4a). Across all subregions (Figure 4c), slight increases represent the dominant trend type for the stable forest, with proportions exceeding 50% in each region.

The NPP trend in the changing forest is more complex. In the changing forest (Figure 4b), 56.9% of the area shows an increasing NPP trend, with 23.8% experiencing a significant increase, primarily concentrated in southwestern and northwestern Yunnan. Slight increases account for 33.1%, distributed in a scattered pattern. NPP decreases occur in 43.1% of the changing forest area, with slight decreases accounting for 29.7%, mainly in the high‐altitude regions of northwestern Yunnan. Areas with significant decreases represent 13.4%, also located in northwestern Yunnan. In the subregions (Figure 4d), slight decreases dominate in central, northwestern, and southeastern Yunnan, while slight increases are predominant in northeastern and southwestern Yunnan.

Overall, the proportion of decreasing NPP in the changing forest is significantly higher than in the stable forest. The area fractions of both significant increases and significant decreases are also larger in the changing forest, indicating that NPP in this forest type exhibits greater variability and stronger spatial heterogeneity.

### 3.4 Future Trends of Forest NPP

**FIGURE 5:**
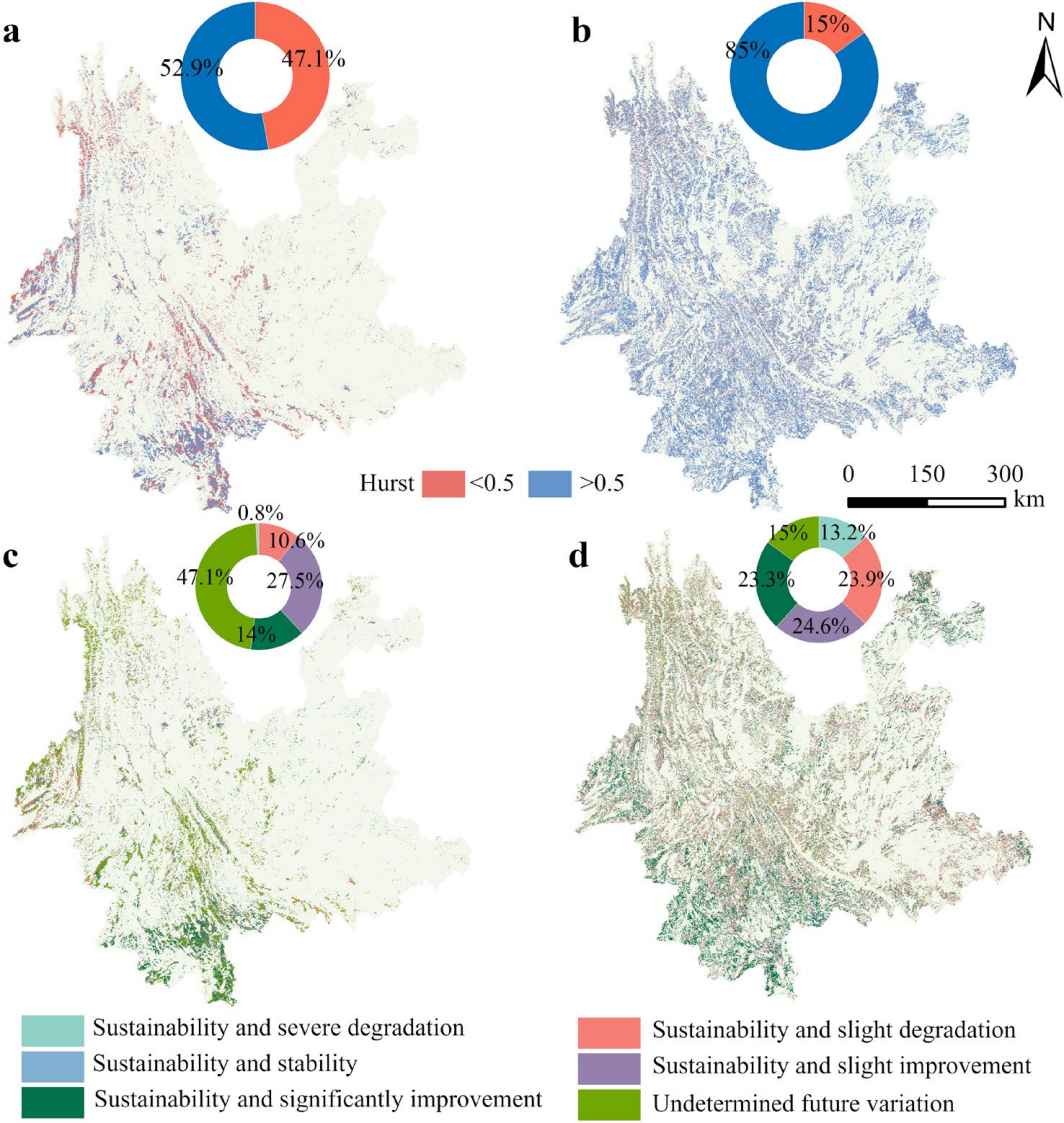
(a, b) Hurst index of the stable forest and the changing forest NPP, and (c, d) future trends of the stable forest and the changing forest NPP, respectively.

Based on the Hurst index analysis results (Figure 5a,b), the NPP time series of the stable forest and the changing forest exhibit long‐term persistence characteristics, with average *H* values of 0.51 and 0.61, respectively. This suggests that the NPP change trends of both forest types are likely to continue following historical patterns in the future.

In the stable forest, regions with *H* < 0.5 account for approximately 47.1%, primarily located in the northern part of southwestern Yunnan and northwestern Yunnan. This suggests that NPP in these areas may experience a trend reversal in the future. Regions with *H* > 0.5 make up 52.9%, mainly concentrated in the southern part of southwestern Yunnan, indicating that NPP in these areas is more likely to continue in the current direction. Based on trend analysis (Figure 5c), about 47.1% of the stable forest area shows uncertainty in the future direction of change, and this spatial distribution overlaps significantly with the low *H*‐value areas. Approximately 41.5% of the area exhibits a persistent increasing trend, mainly concentrated in the southern part of southwestern Yunnan, while the remaining areas are smaller and scattered in distribution.

In the changing forest, regions with *H* < 0.5 account for 15%, mainly distributed in northwestern Yunnan. Regions with *H* > 0.5 account for 85% and are widely distributed across the province. Combined with trend analysis (Figure 5d), approximately 47.9% of the changing forest area is likely to experience a continued improvement in NPP in the future, mainly in southwestern and northeastern Yunnan. About 23.9% of the area shows a persistent mild decrease, primarily in southeastern Yunnan, while the remaining areas exhibit various combinations of trend characteristics.

### 3.5 Analysis of Drivers of Forest NPP Changes at Multiple Spatial Scales

#### 3.5.1 Total Effects

This study used random forest modeling combined with SHAP value analysis to identify and quantify the main drivers of changes in forest NPP. Model accuracy is detailed in Table S4. The results indicate that the dominant drivers of NPP vary significantly across different regions.

**FIGURE 6:**
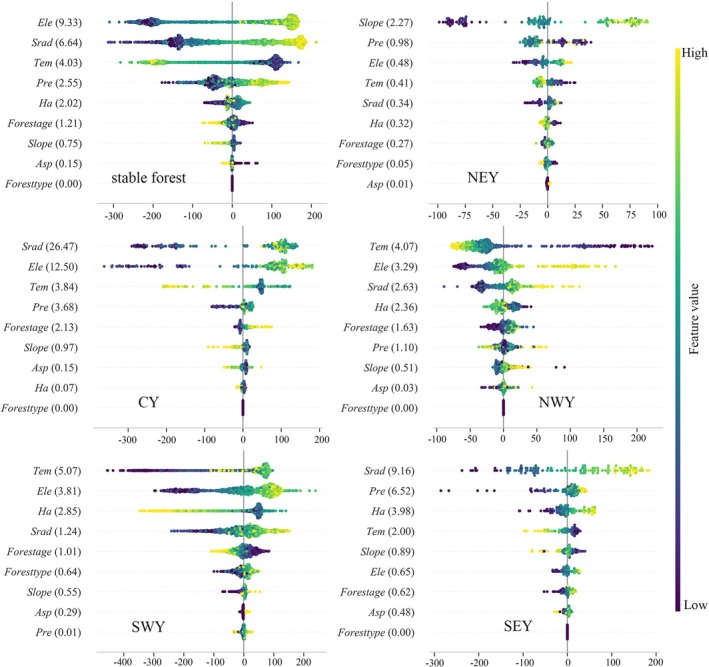
Distribution of SHAP values for the total effect of forest NPP drivers in the stable forest (SF). The figure illustrates the distribution of SHAP values for each feature on forest NPP, where each point represents a sample and the color gradient indicates the magnitude of the feature value (yellow for high values and blue for low values). Features are ordered from high to low by their mean absolute SHAP values (values labeled on the right), with the positive or negative direction of the SHAP value reflecting a positive or negative impact on the forest NPP. All analyses are based on the normalized results of the mean absolute SHAP values, and all subsequent SHAP analyses are plotted or labeled in this way.

In the stable forest (Figure 6), *Ele* is the primary factor influencing the spatial distribution of NPP. Subregional analysis shows that NPP in northeastern Yunnan is mainly controlled by *Slope*, reflecting a terrain‐dominated pattern. In central and southeastern Yunnan, *Srad* is the main driver of NPP. In northwestern and southwestern Yunnan, NPP is primarily influenced by *Tem*. Except for southwestern Yunnan, *Pre* plays an important regulatory role in the other four subregions. In contrast, *Forest type* has a relatively low overall contribution to NPP, with its influence mainly limited to southwestern Yunnan.

**FIGURE 7:**
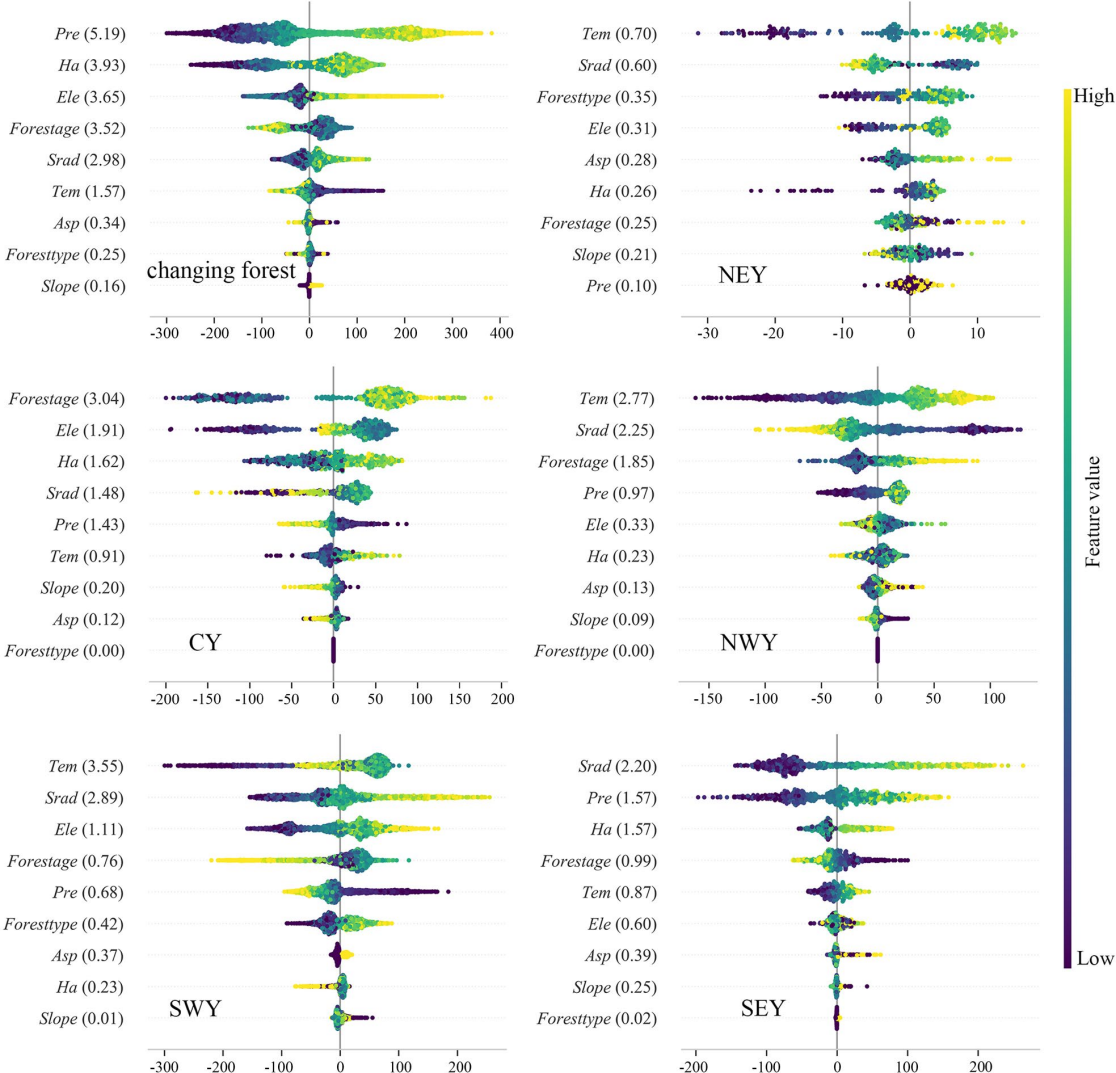
The changing forest SHAP values.

In the changing forest (Figure 7), *Pre* is the most important factor influencing overall NPP variability. Subregional analysis further shows that NPP changes in northeastern, northwestern, and southwestern Yunnan are mainly driven by *Tem*. In southeastern Yunnan, NPP is primarily controlled by *Srad*, while in central Yunnan, *Forest age* is the dominant driver. Compared with the stable forest, the influence of *Ha* is significantly enhanced in the changing forest, emerging as a secondary driver at the overall scale, reflecting the prominent impact of human activities on these ecosystems.

#### 3.5.2 Main Effects

**FIGURE 8:**
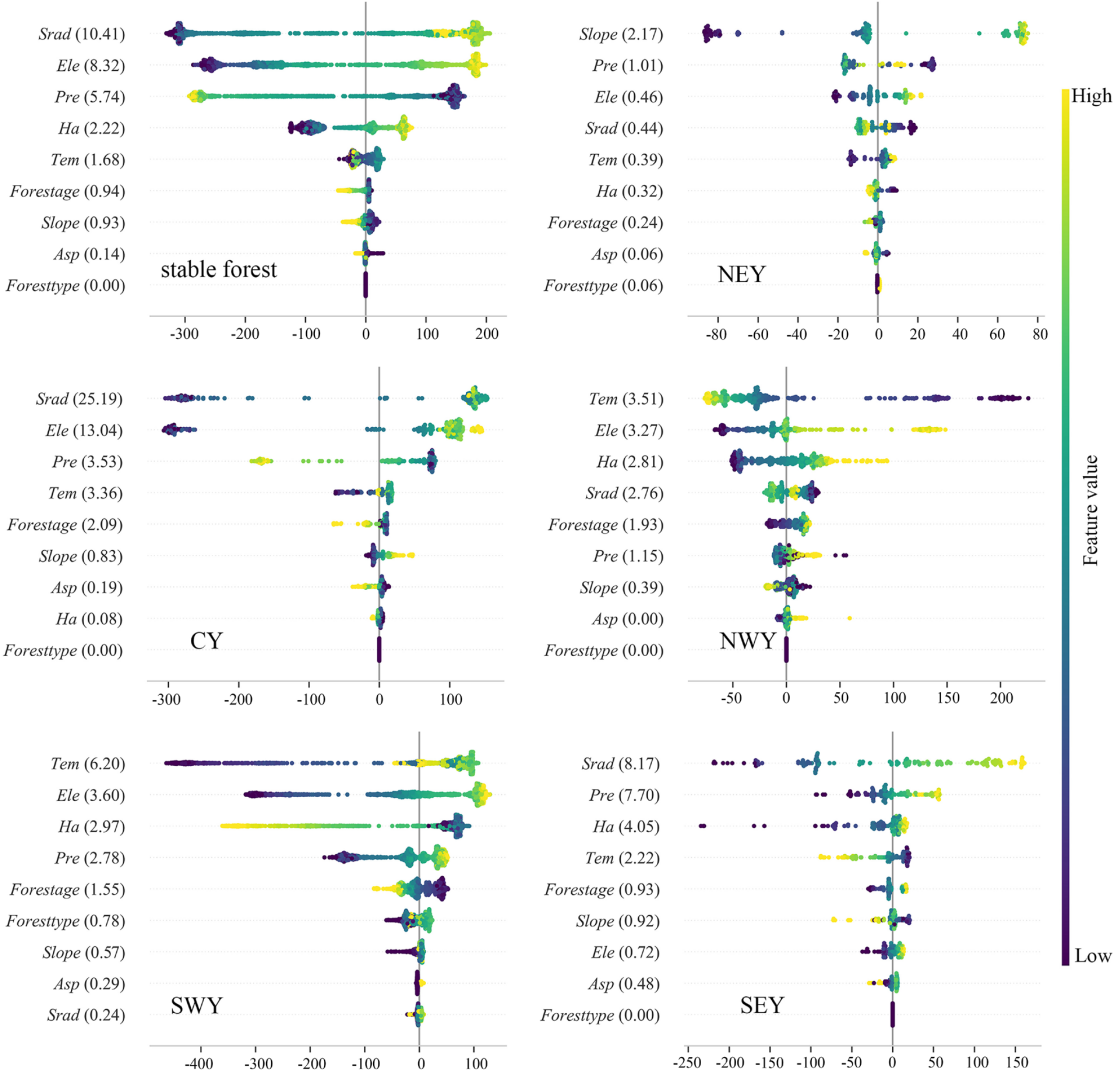
Main effects analysis of the stable forest NPP drivers.

**FIGURE 9:**
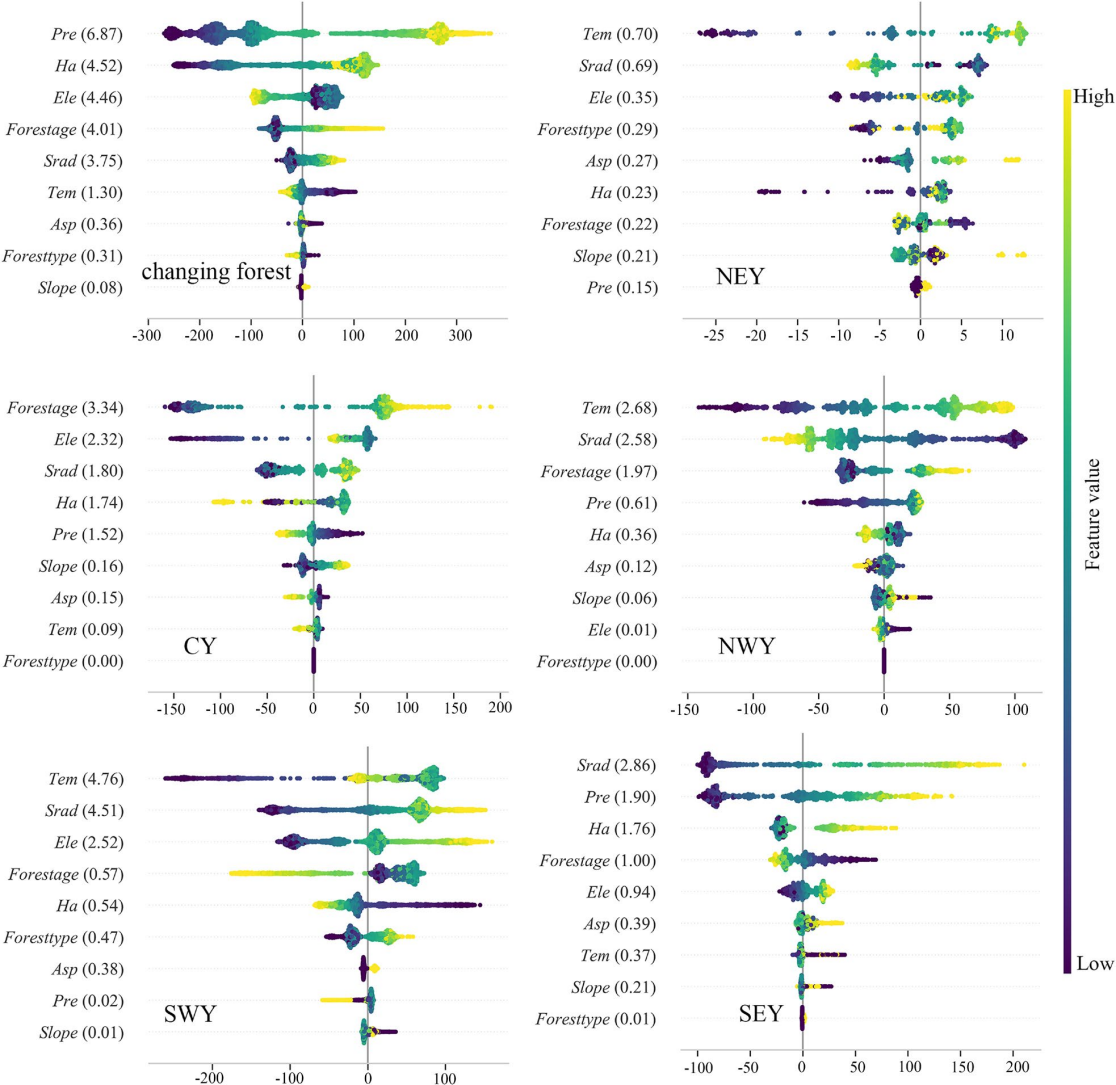
Main effects analysis of the changing forest NPP drivers.

This study further quantified the main effects of each driving variable on forest NPP using the SHAP method, which represents the independent contribution of a single variable to NPP changes while controlling for the influence of other variables. The results (Figures 8 and 9) show that the average SHAP main effect values of most driving variables are lower than their total effect values, indicating significant interactions among variables. Consequently, there are notable differences in the ranking of variable importance based on main effects versus total effects.

In the stable forest (Figure 8), NPP changes are overall primarily controlled by *Srad*. The dominant drivers vary significantly across subregions. In central and southeastern Yunnan, NPP is mainly influenced by *Srad*. In northwestern and southwestern Yunnan, NPP is primarily regulated by *Tem*. In northeastern Yunnan, NPP is mainly controlled by the *Slope*. *Asp* has a relatively weak explanatory power for NPP changes across all subregions.

In the changing forest (Figure 9), NPP changes at the overall scale are primarily driven by *Pre*, with the explanatory power of *Ha* also enhanced. Subregional analysis shows that NPP in northeastern, northwestern, and southwestern Yunnan is mainly controlled by *Tem*. In southeastern Yunnan, NPP is primarily influenced by *Srad*, while in central Yunnan, changes in NPP are dominated by *Forest age*. Overall, the spatiotemporal variation of forest NPP in Yunnan Province is mainly driven by climatic factors, highlighting the central role of climate change in regulating regional ecosystem productivity.

#### 3.5.3 Interaction Analysis

**FIGURE 10:**
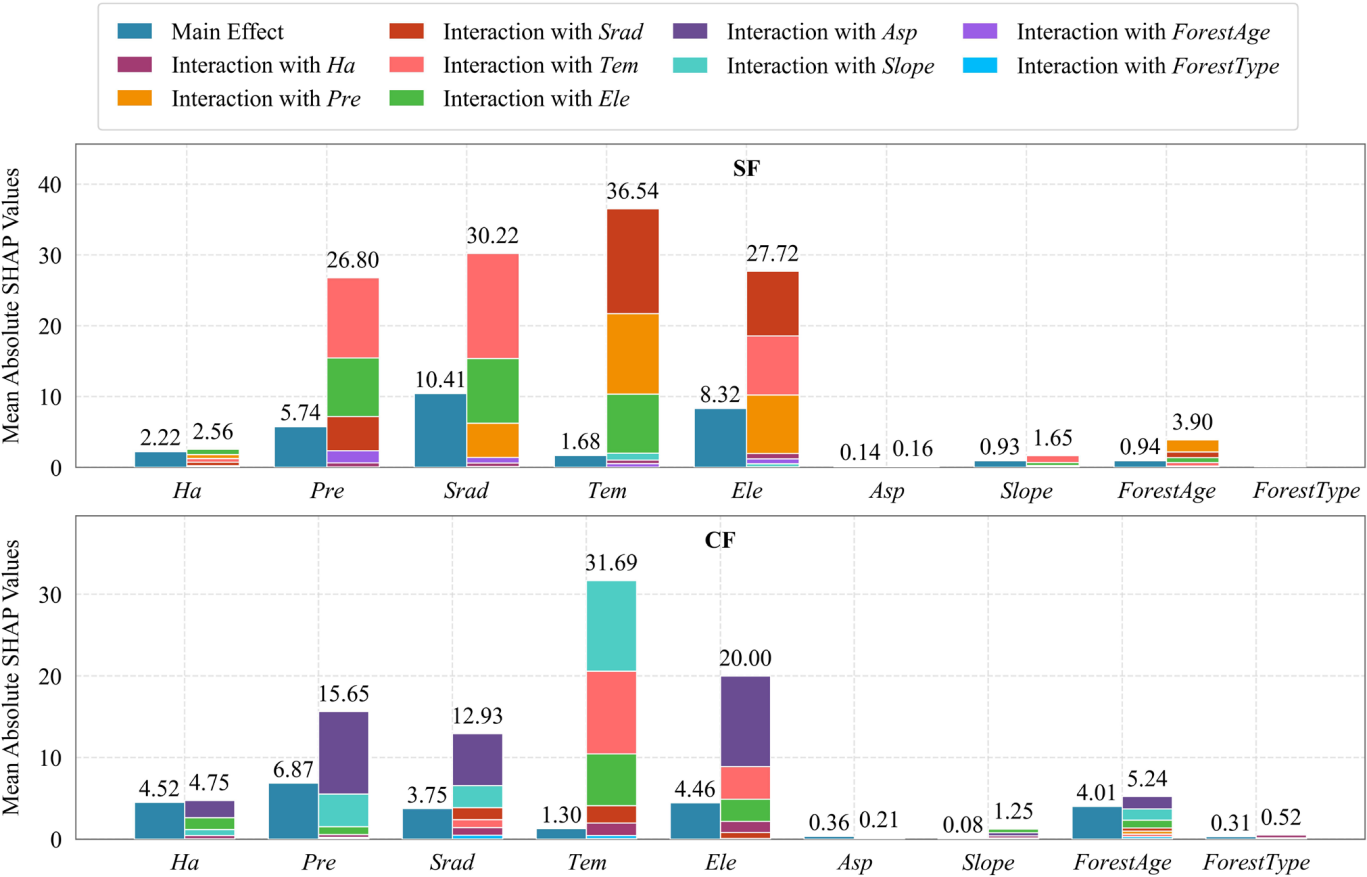
Comparative analysis of main and interaction effects of SF and CF.

The interactive effects of drivers on NPP changes in different forest regions of Yunnan Province exhibit pronounced spatial heterogeneity. Overall analysis (Figure 10 and Figures S1–S5) shows that the interaction effects among factors are generally greater than their individual main effects. This indicates that NPP changes are primarily driven by the synergistic action of multiple factors rather than the independent influence of a single factor.

**FIGURE 11:**
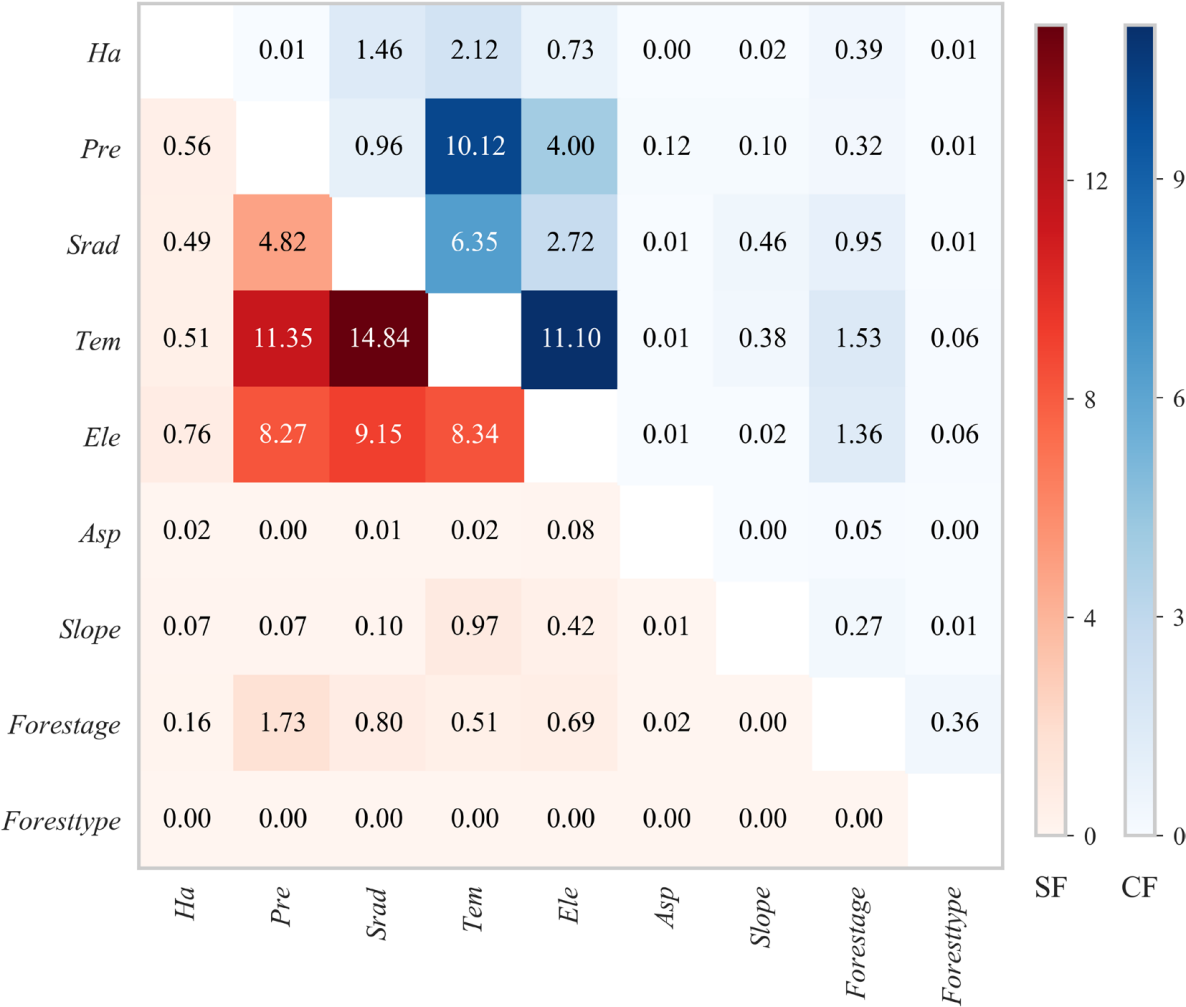
Heatmaps of second‐order interaction effects for SF (lower left) and CF (upper right).

In the stable forest (Figure 11, Figure S6), the interaction between *Tem* and *Srad* exerts a dominant influence on NPP changes at the provincial scale. Subregional analysis shows that NPP changes in northeastern, northwestern, southwestern, and southeastern Yunnan are mainly driven by the interaction between *Pre* and *Srad*. In central Yunnan, NPP changes are primarily influenced by the interaction between *Tem* and *Ele*. Additionally, in southwestern Yunnan, NPP changes are also significantly affected by the interaction between *Tem* and *Srad*.

In the changing forest (Figure 11, Figure S7), NPP changes at the provincial scale and in central Yunnan are primarily driven by the interaction between *Tem* and *Ele*. The dominant interaction types vary significantly across subregions. In northeastern Yunnan, NPP changes are mainly influenced by the combined effects of *Srad* and *Ele*, as well as *forest type* and *forest age*. In central Yunnan, NPP changes are primarily controlled by the interaction between *Tem* and *Srad*. In northwestern Yunnan, the interaction between *Ele* and *Srad* dominates NPP changes. In southwestern and southeastern Yunnan, NPP changes remain primarily governed by the *Tem*‐*Ele* interaction. Apart from these key interactions, the contributions of other variable interactions to NPP changes are relatively limited in most areas.

Overall analysis indicates that the spatiotemporal variation of forest NPP in Yunnan Province is primarily driven by the interaction of multiple factors. The dominant interaction patterns vary significantly among different forest types and geographic subregions, demonstrating that forest ecosystem responses to driving factors are highly context‐dependent.

### 3.6 Threshold Effect of Driving Factors on Forest NPP

This study explored the response thresholds of NPP changes to driving factors using the RF model. In the analysis, the density of peaks along the X‐axis visually reflects the distribution of data points, with dense areas indicating concentrated points and sparse areas indicating fewer points. Considering the potential uncertainty of trend lines in sparse data regions, these areas were not analyzed in depth. To focus on the dominant factors, we selected the six variables with the highest total effect values within each region for discussion.

**FIGURE 12:**
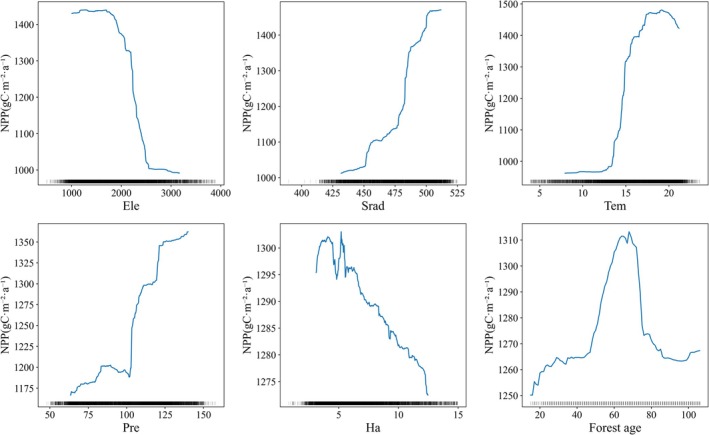
Partial dependence of the stable forest annual mean NPP on each driver.

In the stable forest (Figure 12), NPP responses to driving factors exhibit clear threshold characteristics. *Tem* has an optimal range for NPP, increasing noticeably as *Tem* rises from 14°C to 20°C, but declining above 20°C. *Ele* is negatively correlated with NPP, with NPP continuing to decrease when *Ele* exceeds 2000 m. Solar radiation positively promotes NPP, while *Pre* between 100 and 125 mm significantly enhances it. *Forest age* strongly promotes NPP between 50 and 70 years, but NPP declines when age exceeds 70 years. *Ha* is negatively correlated with NPP. Subregional analysis indicates that although specific response thresholds vary across subregions (Figures S8–S12), the overall response pattern to *Ele* is similar. In southeastern Yunnan, NPP shows a decline when the *Slope* exceeds 25°, representing a distinct local response pattern.

**FIGURE 13:**
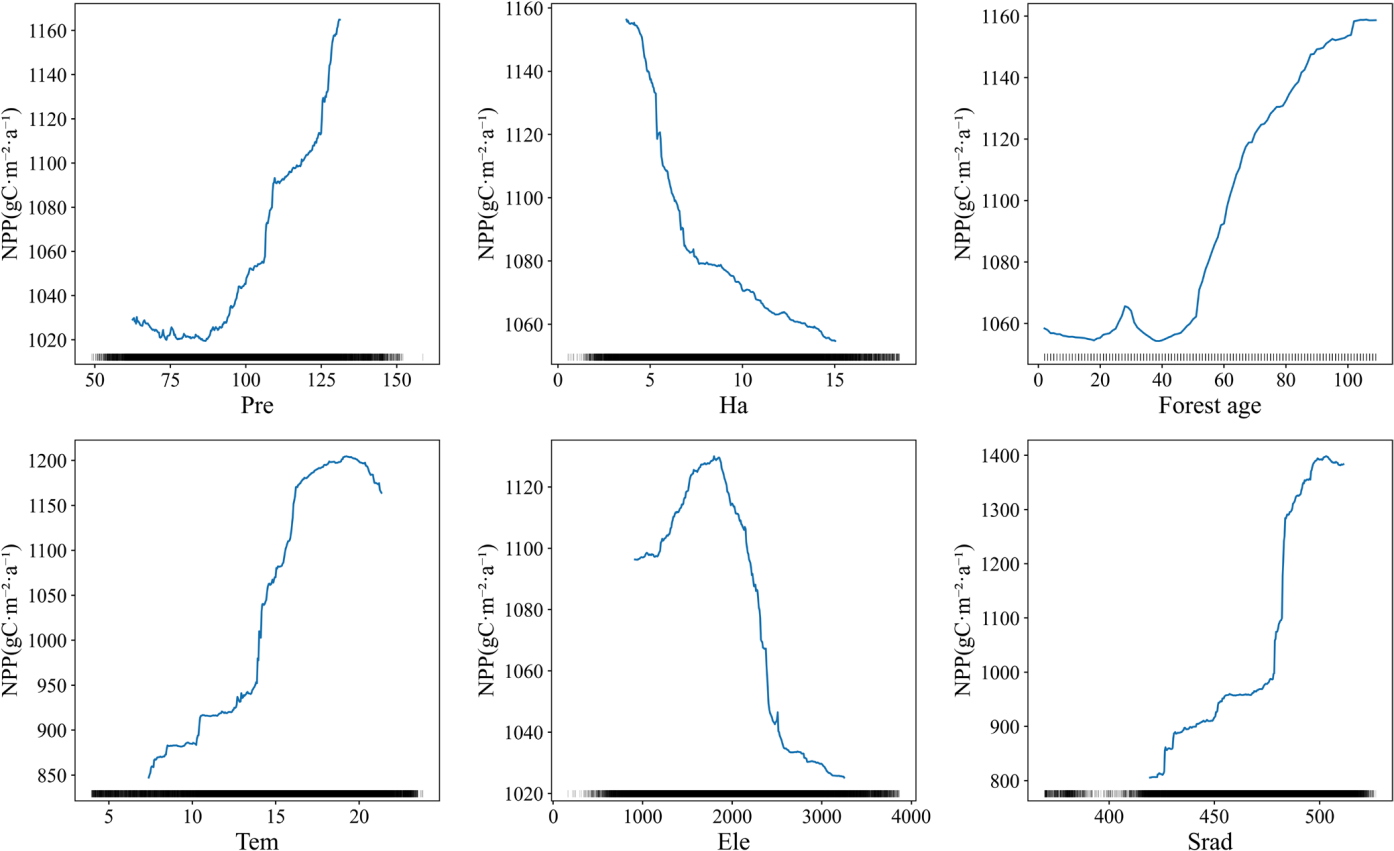
Partial dependence of the changing forest annual mean NPP on each driver.

In the changing forest (Figure 13), NPP responses to driving factors exhibit both commonalities and differences. Common patterns include a significant increase in NPP when *Pre* ranges from 100 to 125 mm, and a negative impact of *Ha* on NPP. *Forest age* continuously promotes NPP between 50 and 100 years. Regarding differences, NPP rises steadily with *Tem* between 10°C and 20°C. *Ele* is positively correlated with NPP in the 1000–2000 m range but becomes negatively correlated above 2000 m. The promoting effect of solar radiation on NPP is mainly concentrated in the 450–500 W/m^2^ range. Subregional comparisons indicate that although response thresholds vary, the overall response patterns are relatively consistent (Figures S13–S17). Northeastern Yunnan shows a unique aspect response, with NPP negatively correlated with *Asp* between 100° and 250°, turning positive above 250°. In northeastern and northwestern Yunnan, NPP is positively correlated with *Ha*, contrary to the overall pattern. Southeastern Yunnan exhibits a distinct *Ele* response: NPP decreases with *Ele* between 1000 and 1700 m, but increases between 1700 and 2000 m.

## 4 Discussion

### 4.1 Differential Response Mechanisms and Pattern Drivers Under the Stable‐Changing Forest Framework

The stable‐changing forest analysis framework developed in this study effectively reveals systematic differences between the two forest types in NPP dynamics, driving mechanisms, and future trends. Spatially, forest NPP in Yunnan Province exhibits an overall pattern of higher values in the south and lower values in the north, closely matching the southwest‐to‐northeast decreasing precipitation gradient (Figure S18a; Chen and Zhang 2023). This pattern reflects the combined influence of the monsoon climate and complex topography.

In the stable forest, NPP changes are mainly characterized by slight improvement (He et al. 2022), with slight increases accounting for 60.1%, showing an overall stable and moderately positive trend consistent with the natural fluctuation patterns of mature ecosystems (Zhang and Zhang 2024). Although future trends are generally persistent (regions with *H* > 0.5 account for 52.9%), nearly half of the area has the potential for trend reversal, indicating a complex response to environmental changes. Analysis of driving mechanisms shows that elevation, as an integrated indicator of water and thermal conditions, is the primary factor controlling spatial variation in NPP (Girardin et al. 2010). At the regional level, NPP increases in northwestern Yunnan are mainly driven by increased precipitation and enhanced solar radiation. In northeastern, southeastern, central, and southwestern Yunnan, NPP changes are more strongly promoted by the synergistic effects of increased precipitation and rising temperature (Figure S18).

In contrast, NPP dynamics in the changing forest exhibit pronounced polarization and clear signatures of human disturbance. The proportions of areas with significant increases (23.8%) and significant decreases (13.4%) are much higher than in the stable forest, reflecting differential responses caused by anthropogenic activities. Future trends show stronger overall persistence, with regions having *H* > 0.5 reaching 85%, indicating that large‐scale improvement and degradation trends may continue over the long term. This persistence does not imply ecosystem stability but highlights that carbon sink functions will face greater divergence and higher uncertainty in the future. Regarding driving mechanisms, the dominant factor shaping the overall pattern in the changing forest has shifted from elevation, as in the stable forest, to precipitation. Increases in precipitation, together with ecological projects such as returning farmland to forest (Li, Wang, Chen, and Zhang 2022) and rocky desertification control (Xiao and Xiong 2022), jointly drive NPP increases. This demonstrates that land‐use changes, as strong ecological disturbances, have reshaped the original ecological balance, making forests more sensitive and vulnerable to both climatic factors and human management (Stritih et al. 2021).

### 4.2 Scale Effects and Interactions of Dominant Drivers

This study confirms that climatic factors are the core drivers regulating forest NPP at the regional scale in Yunnan Province (Ji et al. 2020). SHAP analysis further reveals the underlying mechanisms of their influence. For example, the dominant role of precipitation in the changing forest may be related to the incomplete root development in newly afforested or disturbed forest areas, making these regions more sensitive to fluctuations in water availability (van Meerveld and Seibert 2025).

The results indicate that driving mechanisms exhibit significant scale effects. At the provincial scale, elevation and precipitation are the primary drivers of NPP in the stable forest and the changing forest, respectively. At the subregional scale, dominant factors diverge, including temperature, slope, and forest age (Li, Wang, Liu, et al. 2022). This suggests that broad‐scale general patterns are often modified locally by specific topography‐climate combinations. For example, in the stable forest, low‐temperature constraints in northwestern Yunnan and steep‐slope limitations in northeastern Yunnan replace elevation as the dominant factor. Ignoring such spatial heterogeneity may increase uncertainty in regional carbon sink assessments.

Another key finding is that the total effects of driving factors on NPP are primarily derived from their interactions rather than their individual main effects. The SHAP interaction matrix shows that for core factors such as temperature, elevation, and precipitation, the strength of their interactions often approaches or exceeds their own main effects. For example, in the changing forest, the interaction between temperature and elevation is markedly stronger than the main effect of elevation alone. This indicates that the influence of temperature on NPP is largely mediated through its synergistic interaction with elevation. In low‐elevation areas, warming may promote vegetation growth, whereas at high elevations, the same temperature increase can suppress NPP due to enhanced water stress or freeze–thaw cycles (Li, Zhou, et al. 2025; Zhang et al. 2022).

### 4.3 Ecological Implications of Nonlinear Responses and Threshold Behavior

This study identifies nonlinear responses and threshold behaviors for several key driving factors. NPP peaks when forest age is 50–70 years and declines thereafter, reflecting the life‐cycle characteristic of increased maintenance respiration as forests transition from maturity to over‐maturity (Besnard et al. 2018; Pregitzer and Euskirchen 2004). NPP declines significantly when the slope exceeds 25°, directly associated with shallow soils and poor water and nutrient retention on steep slopes (Duan et al. 2016). Temperature shows a typical unimodal effect on NPP, with an optimal range of 14°C–20°C, consistent with enzyme kinetics principles in plant physiological ecology (Liu, Chen, et al. 2025). At low temperatures, biochemical reaction rates are limited, whereas photosynthetic efficiency is maximized within the optimal range. Beyond the threshold, thermal stress from enhanced photorespiration and reduced enzyme activity becomes the dominant control (Chen, Wang, et al. 2023; Yvon‐Durocher et al. 2012). These well‐defined thresholds provide quantitative indicators for ecosystem monitoring and management, serving as valuable references for ecological early warning.

Notably, in northeastern and northwestern Yunnan, NPP is positively correlated with human activity intensity. Given that the human activity index used in this study primarily reflects development intensity, this relationship is more likely due to the spatial overlap between human activities and areas with favorable natural conditions (Chen, Ma, et al. 2023). Human settlements and agriculture typically occur in regions with suitable water and heat conditions and high vegetation growth potential, resulting in a strong spatial correspondence. Therefore, this positive correlation likely reflects human preferential selection rather than a direct enhancement of NPP.

### 4.4 Limitations and Prospects

The limitations of this study arise mainly from the data and the models. First, the analysis relies entirely on remote sensing data and lacks direct ground observations, so the results retain a degree of uncertainty. Second, the analysis of driving factors is constrained by data availability, and we can only distinguish between natural forests and plantations rather than identify specific plantation types such as eucalyptus or rubber. The human activity index also cannot capture differences in management practices, such as fertilization or thinning. Finally, although RF and SHAP describe complex nonlinear relationships, they remain statistical learning methods and cannot explain the biophysical mechanisms of NPP change in the same way as process‐based ecological models.

Future studies could combine forest inventories, flux tower measurements, and long‐term monitoring at representative sites to conduct more detailed validation. High‐resolution vegetation structure data from LiDAR can be integrated with management information to assess the effects of different interventions on NPP. In addition, incorporating process‐based ecological models alongside statistical models could provide complementary insights and deepen the understanding of forest productivity changes from a mechanistic perspective.

## 5 Conclusion

This study focuses on forest ecosystems in Yunnan Province, where terrain and climate conditions are highly complex, to examine the spatial heterogeneity of NPP and its driving differences. We developed a comparative framework for the stable forest and the changing forest, divided the study area into five sub‐regions, and applied RF and SHAP methods to analyze the NPP dynamics and driving mechanisms across forest types and regions. The main conclusions are as follows:

1. The increase in NPP within the stable forest is mainly driven by the joint effects of rising precipitation and increasing temperature. In the changing forest, the marked enhancement of NPP is primarily promoted by ecological restoration programs and improved forest management. Model projections show that about 41.5% of the area in the stable forest will maintain continuous improvement, and about 47.9% of the area in the changing forest is also expected to exhibit sustained growth.
2. At the provincial scale, the NPP of both the stable forest and the changing forest is mainly controlled by elevation and precipitation. At the sub‐regional scale, dominant factors shift and include solar radiation, temperature, and forest age, while the influence of human activities becomes markedly stronger in the changing forest. Overall, the interactive effects among multiple factors generally exceed the main effects of individual variables, with temperature showing particularly strong synergistic contributions.
3. Temperature (14°C–20°C), precipitation (100–125 mm), elevation (around 2000 m), and forest age (50–70 years) all exhibit clear optimal response ranges, beyond which the direction of NPP change shifts noticeably. Compared with the stable forest, the changing forest displays broader response ranges, indicating greater environmental tolerance.

Overall, the dynamic response of forest NPP in Yunnan Province exhibits pronounced regional patterns, and these findings provide a scientific basis for precision forest management and carbon‐sink enhancement.

## Author Contributions

**Kun Yang:** conceptualization (equal), funding acquisition (equal), project administration (equal), supervision (equal), writing – review and editing (equal). **Xiaofang Yang:** data curation (equal), formal analysis (equal), methodology (equal), software (equal), validation (equal), visualization (equal), writing – original draft (equal). **Shaohua Zhang:** methodology (equal), software (equal), supervision (equal), writing – review and editing (equal). **Wenxia Zeng:** data curation (equal), investigation (equal), software (equal). **Jing Liu:** data curation (equal), investigation (equal). **Yan Rao:** data curation (equal), investigation (equal). **Yan Ma:** data curation (equal), investigation (equal). **Changyou Bi:** data curation (equal), investigation (equal), software (equal).

## Funding

This study was financially supported by the National Natural Science Foundation of China (grant numbers 42471469, 42361064 and 42530115), the Key Research and Development Program of Yunnan Province No. 202303AC100009, and the Graduate Research and Innovation Fund of Yunnan Normal University (YJSJJ25‐B149). This work was supported by the National Natural Science Foundation of China (grant numbers 42471469 and 42071381), the Key Research and Development Program of Yunnan Province (202303AC100009) and the Graduate Research and Innovation Fund of Yunnan Normal University (YJSJJ25‐B149).

## Conflicts of Interest

The authors declare no conflicts of interest.

## Supporting information


**Table S1:** Forest accuracy validation in the MCD12Q1 dataset.
**Table S2:** The look‐up table of ${\text{NDVI}}_{\max}$, ${\text{NDVI}}_{\min}$, and $\varepsilon^{*}$ values for different land cover types that adopted from (Zhu et al. 2006).
**Table S3:** Classification criteria for future changes in forest NPP trends.
**Table S4:** The simulation accuracy of the random forest model.
**Figure S1:** Comparative analysis of main and interaction effects in stable forest and changing forest regions of northeastern Yunnan.
**Figure S2:** Comparative analysis of main and interaction effects in stable forest and changing forest regions of central Yunnan.
**Figure S3:** Comparative analysis of main and interaction effects in stable forest and changing forest regions of northwestern Yunnan.
**Figure S4:** Comparative analysis of main and interaction effects in stable forest and changing forest regions of southwestern Yunnan.
**Figure S5:** Comparative analysis of main and interaction effects in stable forest and changing forest regions of southeastern Yunnan.
**Figure S6:** Heatmap of second‐order interaction effects among subregions within the stable forest.
**Figure S7:** Heatmap of second‐order interaction effects among subregions within the changing forest.
**Figure S8:** Partial dependence of annual mean forest NPP on driving factors in northeastern Yunnan within the stable forest.
**Figure S9:** Partial dependence of annual mean forest NPP on driving factors in central Yunnan within the stable forest.
**Figure S10:** Partial dependence of annual mean forest NPP on driving factors in northwestern Yunnan within the stable forest.
**Figure S11:** Partial dependence of annual mean forest NPP on driving factors in southwestern Yunnan within the stable forest.
**Figure S12:** Partial dependence of annual mean forest NPP on driving factors in southeastern Yunnan within the stable forest.
**Figure S13:** Partial dependence of annual mean forest NPP on driving factors in northeastern Yunnan within the changing forest.
**Figure S14:** Partial dependence of annual mean forest NPP on driving factors in central Yunnan within the changing forest.
**Figure S15:** Partial dependence of annual mean forest NPP on driving factors in northwestern Yunnan within the changing forest.
**Figure S16:** Partial dependence of annual mean forest NPP on driving factors in southwestern Yunnan within the changing forest.
**Figure S17:** Partial dependence of annual mean forest NPP on driving factors in southeastern Yunnan within the changing forest.
**Figure S18:** Distribution of monthly average precipitation (a), trends in precipitation (b), solar radiation (c), and temperature (d).

## Data Availability

The research data can be accessed at https://doi.org/10.5281/zenodo.17627913.
