# Supplementary material for: Precipitation Dominates Forest Net Primary Productivity Variations With Distinct Regional Differences in Yunnan Province, China
Source: Ecol Evol. 2026 Jan 4;16(1):e72893. doi: 10.1002/ece3.72893 (PMC12765817; doi:10.1002/ece3.72893)
Supplement: Supplementary file 1 — Table S1: Forest accuracy validation in the MCD12Q1 dataset. Table S2: The look‐up table of NDVImax, NDVImin, and ε* values for different land cover types that adopted from (Zhu et al. 2006). Table S3: Classification criteria for future changes in forest NPP trends. Table S4: The simulation accuracy of the random forest model. Figure S1: Comparative analysis of main and interaction effects in stable forest and changing forest regions of northeastern Yunnan. Figure S2: Comparative analysis of main and interaction effects in stable forest and changing forest regions of central Yunnan. Figure S3: Comparative analysis of main and interaction effects in stable forest and changing forest regions of northwestern Yunnan. Figure S4: Comparative analysis of main and interaction effects in stable forest and changing forest regions of southwestern Yunnan. Figure S5: Comparative analysis of main and interaction effects in stable forest and changing forest regions of southeastern Yunnan. Figure S6: Heatmap of second‐order interaction effects among subregions within the stable forest. Figure S7: Heatmap of second‐order interaction effects among subregions within the changing forest. Figure S8: Partial dependence of annual mean forest NPP on driving factors in northeastern Yunnan within the stable forest. Figure S9: Partial dependence of annual mean forest NPP on driving factors in central Yunnan within the stable forest. Figure S10: Partial dependence of annual mean forest NPP on driving factors in northwestern Yunnan within the stable forest. Figure S11: Partial dependence of annual mean forest NPP on driving factors in southwestern Yunnan within the stable forest. Figure S12: Partial dependence of annual mean forest NPP on driving factors in southeastern Yunnan within the stable forest. Figure S13: Partial dependence of annual mean forest NPP on driving factors in northeastern Yunnan within the changing forest. Figure S14: Partial dependence of annual mean forest NPP on driving [file ECE3-16-e72893-s001.docx]

Supplementary Material

**Precipitation dominates forest net primary productivity variations with distinct regional differences in Yunnan Province, China**

Table S1: Forest accuracy validation in the MCD12Q1 dataset.

| Accuracy Metrics | 11 June 2014 | 20 May 2020 |
| --- | --- | --- |
| Overall Accuracy (OA) | 0.870 | 0.872 |
| User's Accuracy (UA) | 0.974 | 0.962 |
| Producer's Accuracy (PA) | 0.867 | 0.881 |

Note: The study established 1,200 validation sample points across both forested and non-forested areas. After comparison with historical imagery from WorldImagery Wayback (https://livingatlas.arcgis.com/wayback) (2014-06-11 and 2020-05-20), the accuracy evaluation results showed user accuracy of 0.97 for 2014 and 0.96 for 2020.

Table S2: The look-up table of $\boldsymbol{NDVI}_{\boldsymbol{max}}$, $\boldsymbol{NDVI}_{\boldsymbol{min}}$, and $\boldsymbol{\varepsilon}^{\boldsymbol{*}}$ values for different land cover types that adopted from (Zhu et al., 2006).

| Vegetation type | ${NDVI}_{max}$ | ${NDVI}_{min}$ | ${SR}_{max}$ | ${SR}_{min}$ | $\varepsilon^{*}$ |
| --- | --- | --- | --- | --- | --- |
| Evergreen Needleleaf Forest | 0.647 | 0.023 | 4.67 | 1.05 | 0.389 |
| Evergreen Broadleaf Forest | 0.676 | 0.023 | 5.17 | 1.05 | 0.985 |
| Deciduous Needleleaf Forest | 0.738 | 0.023 | 6.63 | 1.05 | 0.485 |
| Deciduous Broadleaf Forest | 0.747 | 0.023 | 6.91 | 1.05 | 0.692 |
| Mixed Forest | 0.702 | 0.023 | 4.92 | 1.05 | 0.475 |
| Shrubland | 0.636 | 0.023 | 4.49 | 1.05 | 0.542 |
| Grassland | 0.634 | 0.023 | 4.46 | 1.05 | 0.542 |
| Farmland | 0.634 | 0.023 | 4.46 | 1.05 | 0.542 |
| Built-up land | 0.634 | 0.023 | 4.46 | 1.05 | 0.196 |
| Unused land | 0.634 | 0.023 | 4.46 | 1.05 | 0.217 |

Table S3: Classification criteria for future changes in forest NPP trends.

| Slope (S) | Z-statistic (Z) | Hurst Exponent (H) | Variation types |
| --- | --- | --- | --- |
| <-0.0005 | ≤ -1.96 | >0.5 | Consistent and significant degradation |
| <-0.0005 | -1.96 ~ 1.96 | >0.5 | Consistent and slight degradation |
| -0.0005 ~ 0.0005 | -1.96 ~ 1.96 | >0.5 | Consistent and stable |
| ≥0.0005 | -1.96 ~ 1.96 | >0.5 | Consistent and slight improvement |
| ≥0.0005 | ≥ 1.96 | >0.5 | Consistent and significant improvement |
| - | - | <0.5 | Undetermined future variation trend |

Table S4: The simulation accuracy of the random forest model.

| Region 1 | R^2^ | RMSE | Region 2 | R^2^ | RMSE |
| --- | --- | --- | --- | --- | --- |
| Stable forest | 0.84 | 191.92 | Changing forest | 0.82 | 142.65 |
| NEY | 0.67 | 61.83 | NEY | 0.48 | 53.61 |
| CY | 0.71 | 203.82 | CY | 0.52 | 183.21 |
| NWY | 0.78 | 97.39 | NWY | 0.75 | 88.95 |
| SWY | 0.84 | 165.36 | SWY | 0.66 | 201.63 |
| SEY | 0.66 | 155.15 | SEY | 0.67 | 123.57 |


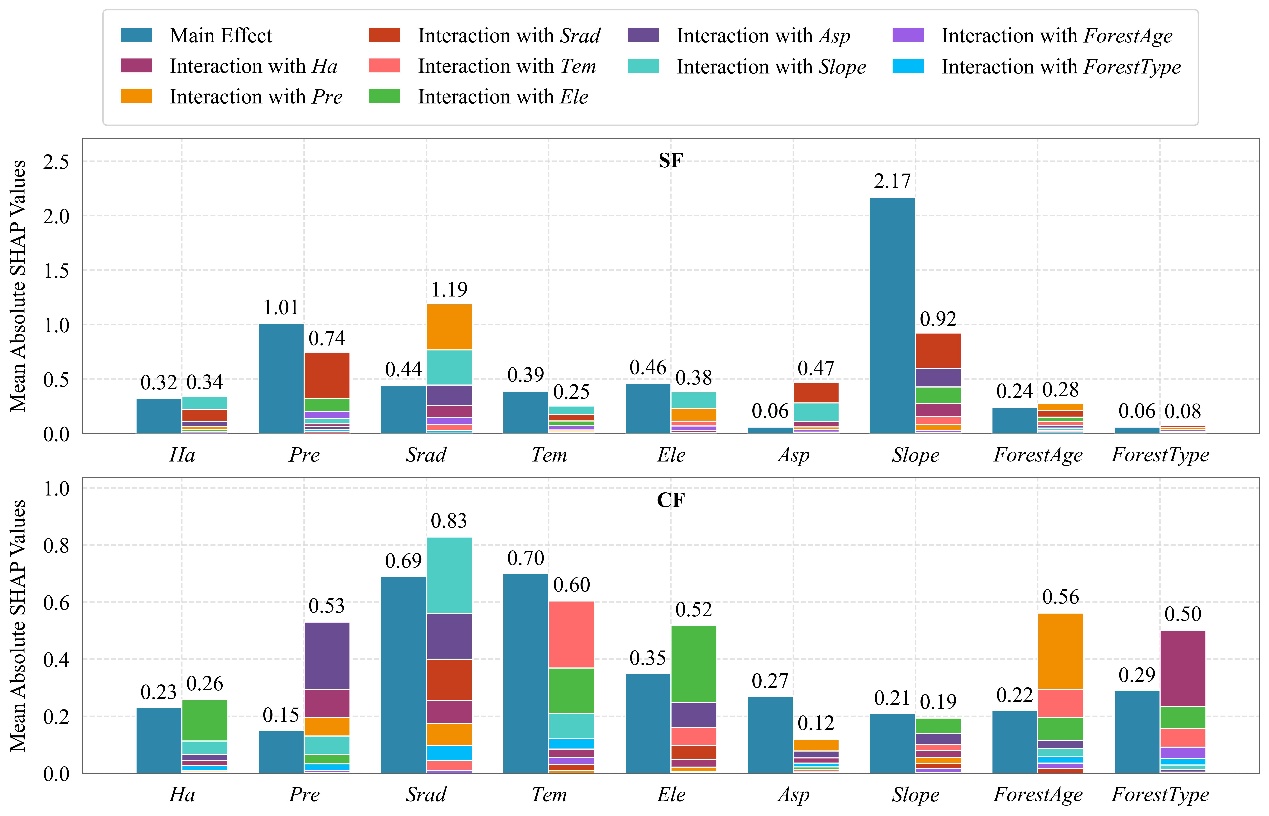


Figure S1: Comparative analysis of main and interaction effects in stable forest and changing forest regions of northeastern Yunnan.


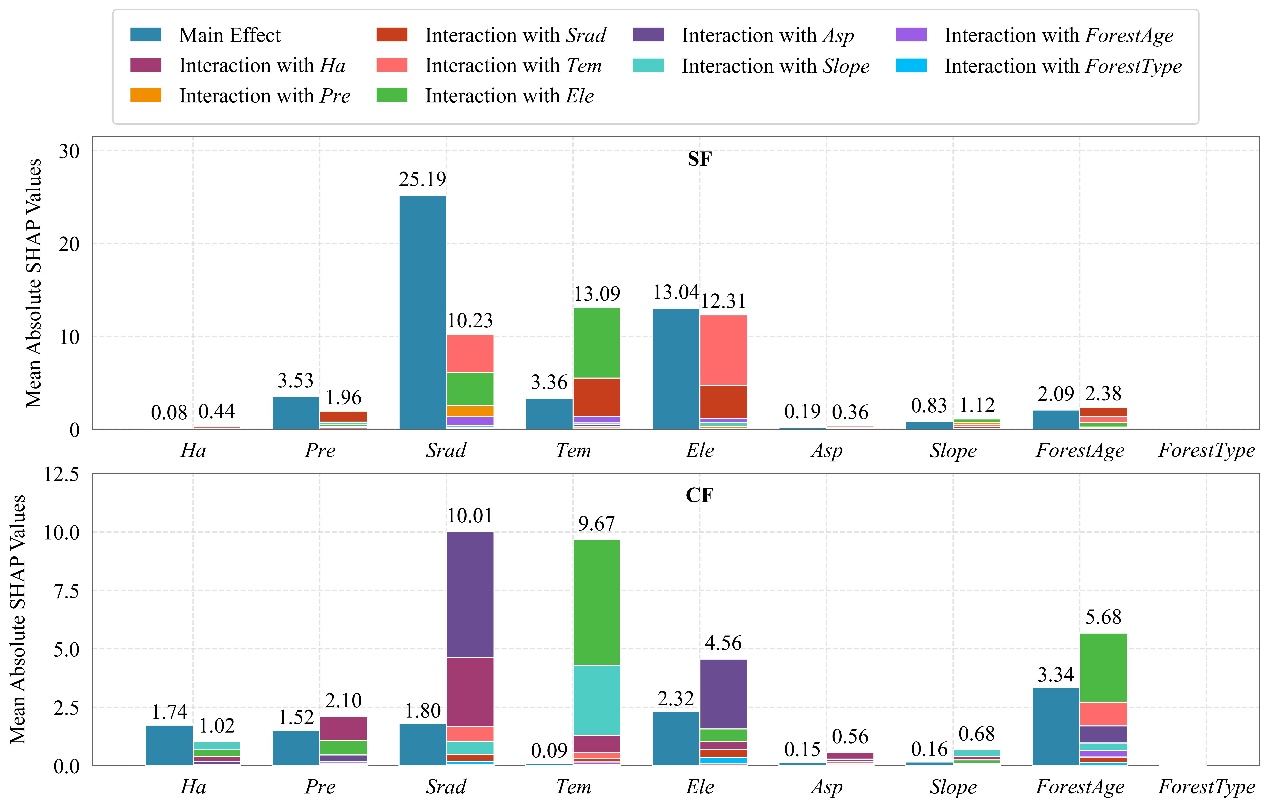


Figure S2: Comparative analysis of main and interaction effects in stable forest and changing forest regions of central Yunnan.


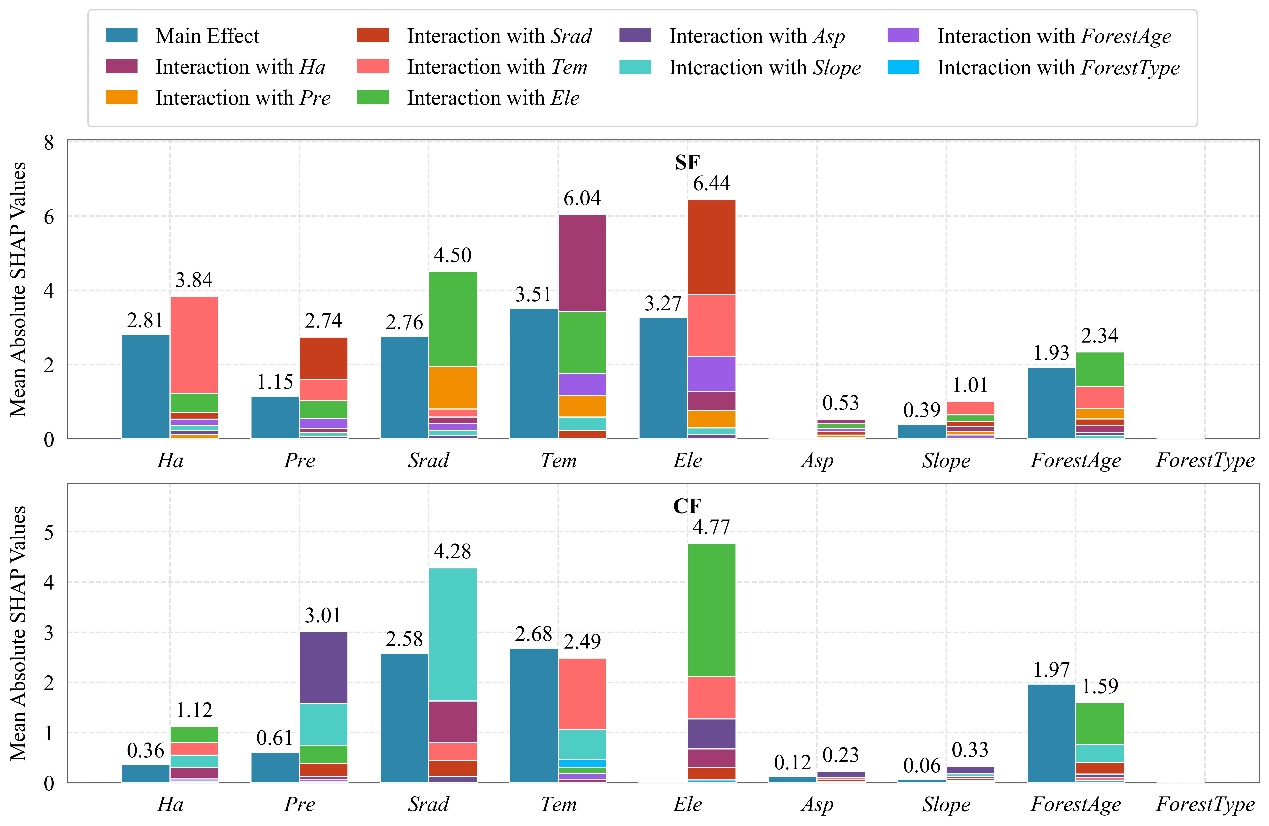


Figure S3: Comparative analysis of main and interaction effects in stable forest and changing forest regions of northwestern Yunnan.


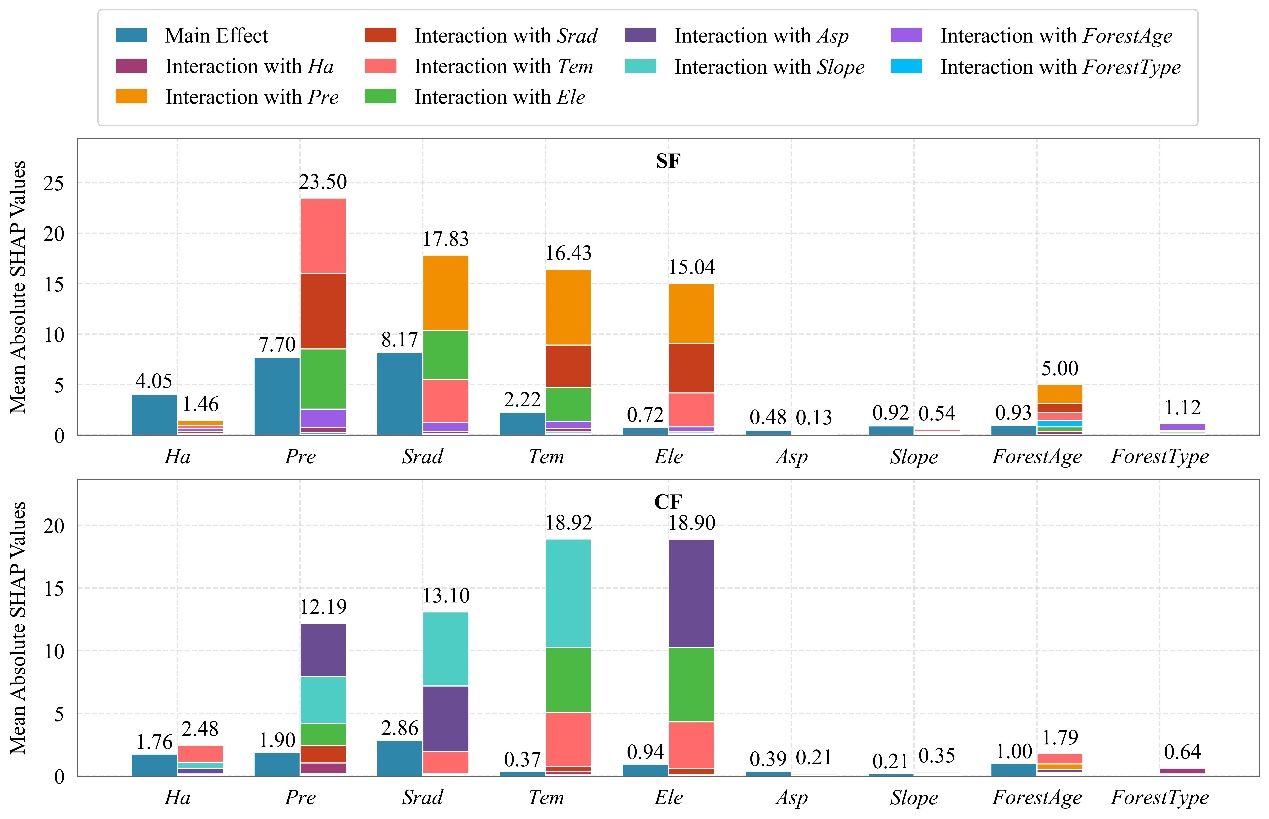


Figure S4: Comparative analysis of main and interaction effects in stable forest and changing forest regions of southwestern Yunnan.


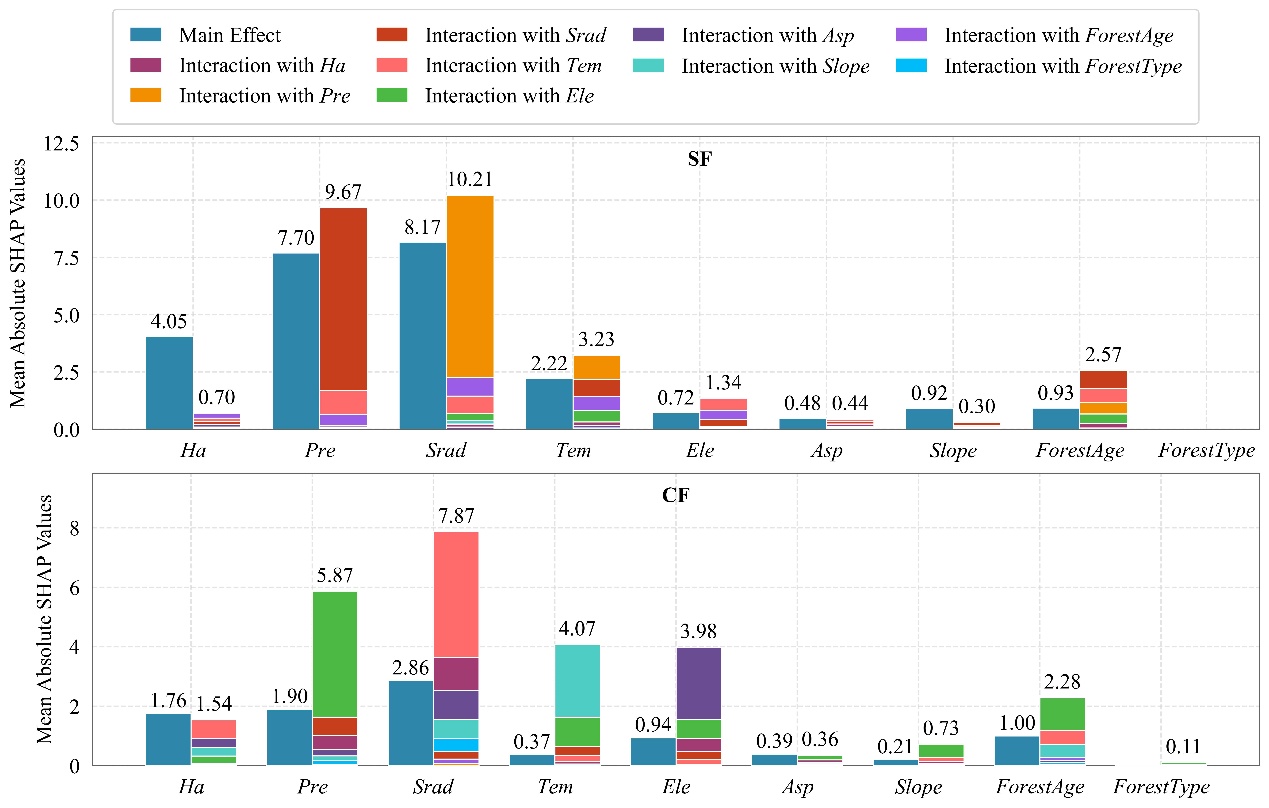


Figure S5: Comparative analysis of main and interaction effects in stable forest and changing forest regions of southeastern Yunnan.


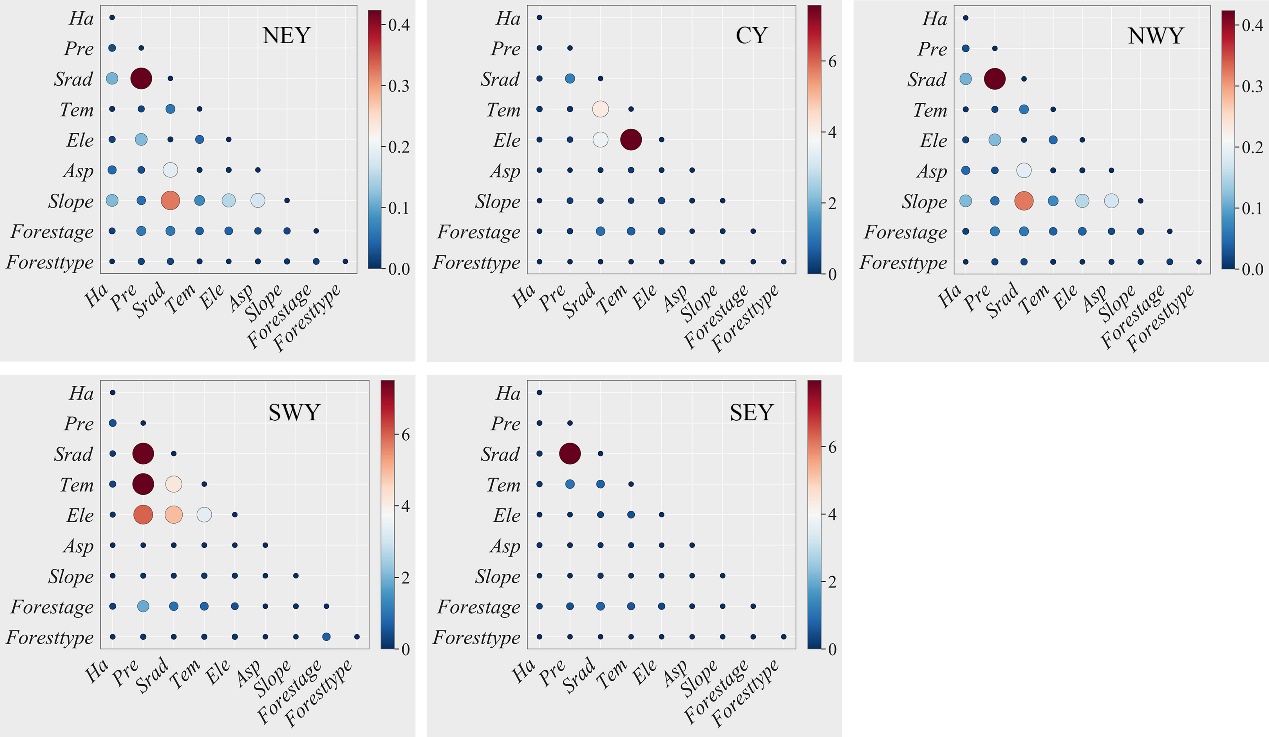


Figure S6: Heatmap of second-order interaction effects among subregions within the stable forest.


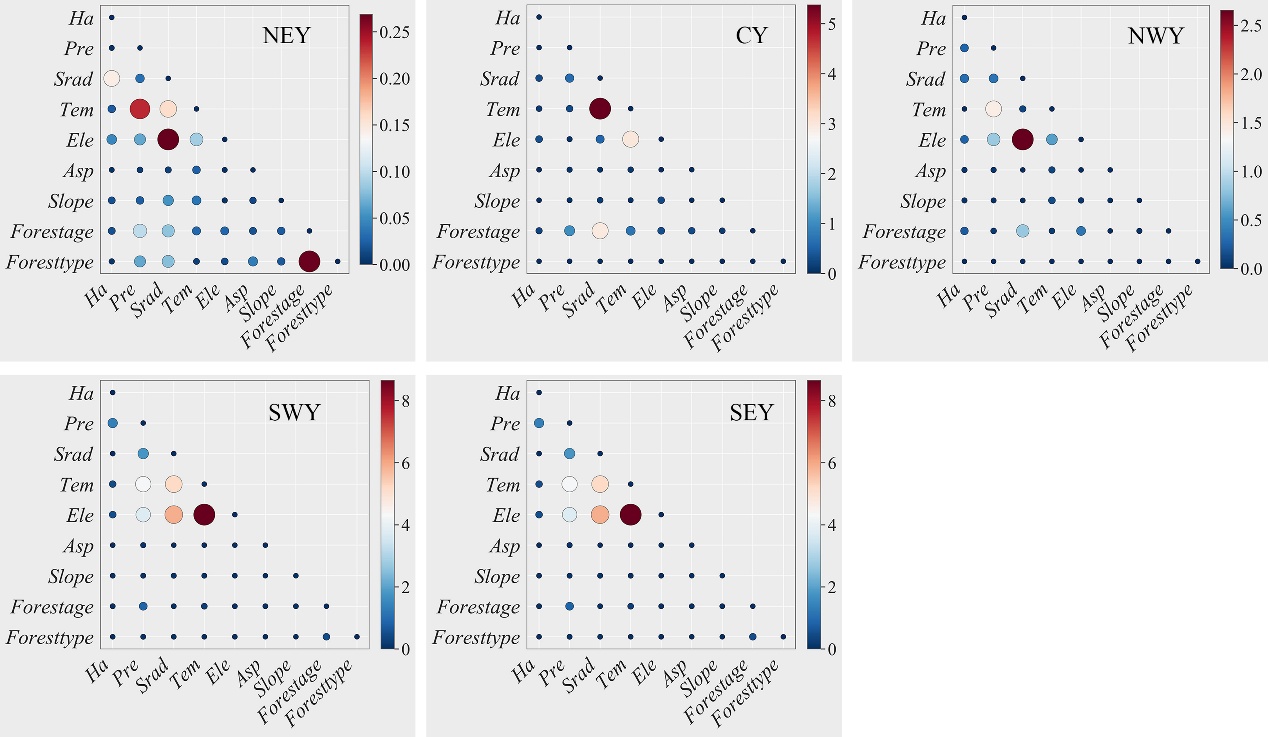


Figure S7: Heatmap of second-order interaction effects among subregions within the changing forest.


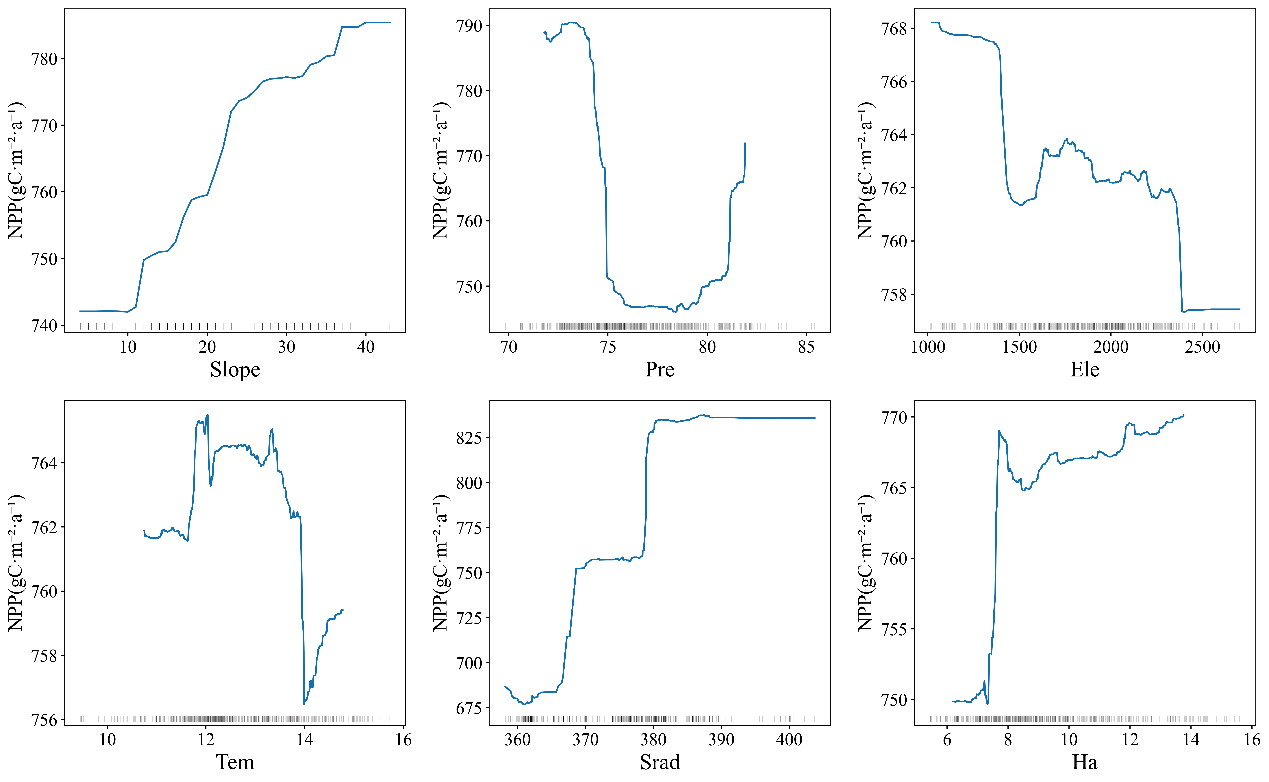


**Figure S8:** Partial dependence of annual mean forest NPP on driving factors in northeastern Yunnan within the stable forest.


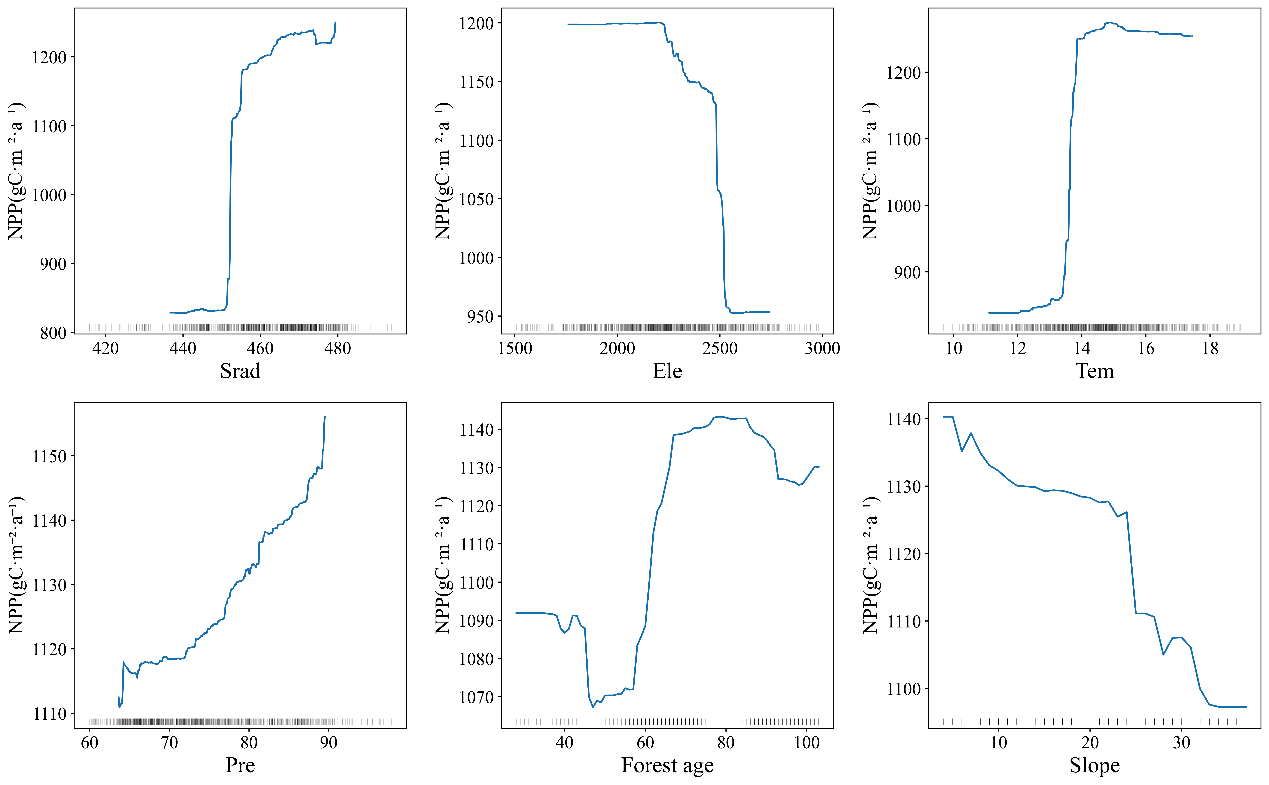


**Figure S9:** Partial dependence of annual mean forest NPP on driving factors in central Yunnan within the stable forest.


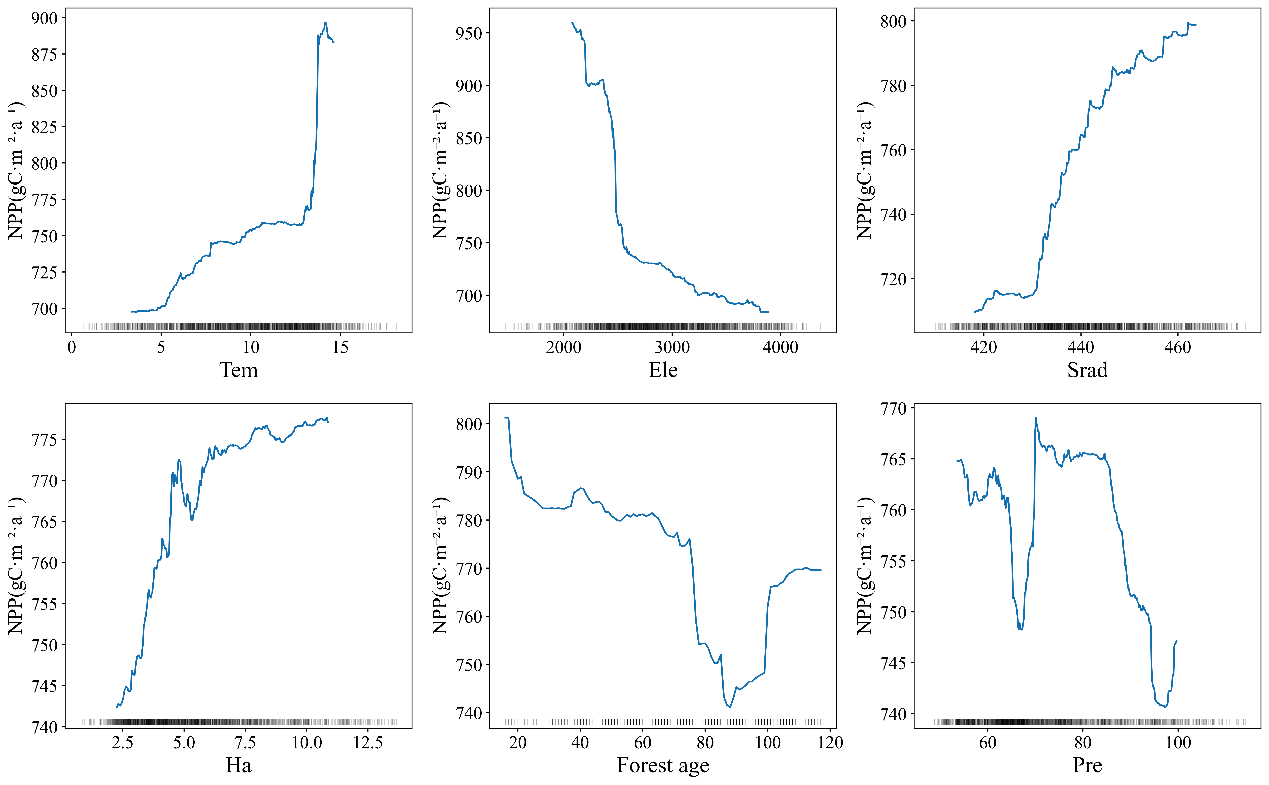


**Figure S10:** Partial dependence of annual mean forest NPP on driving factors in northwestern Yunnan within the stable forest.

**
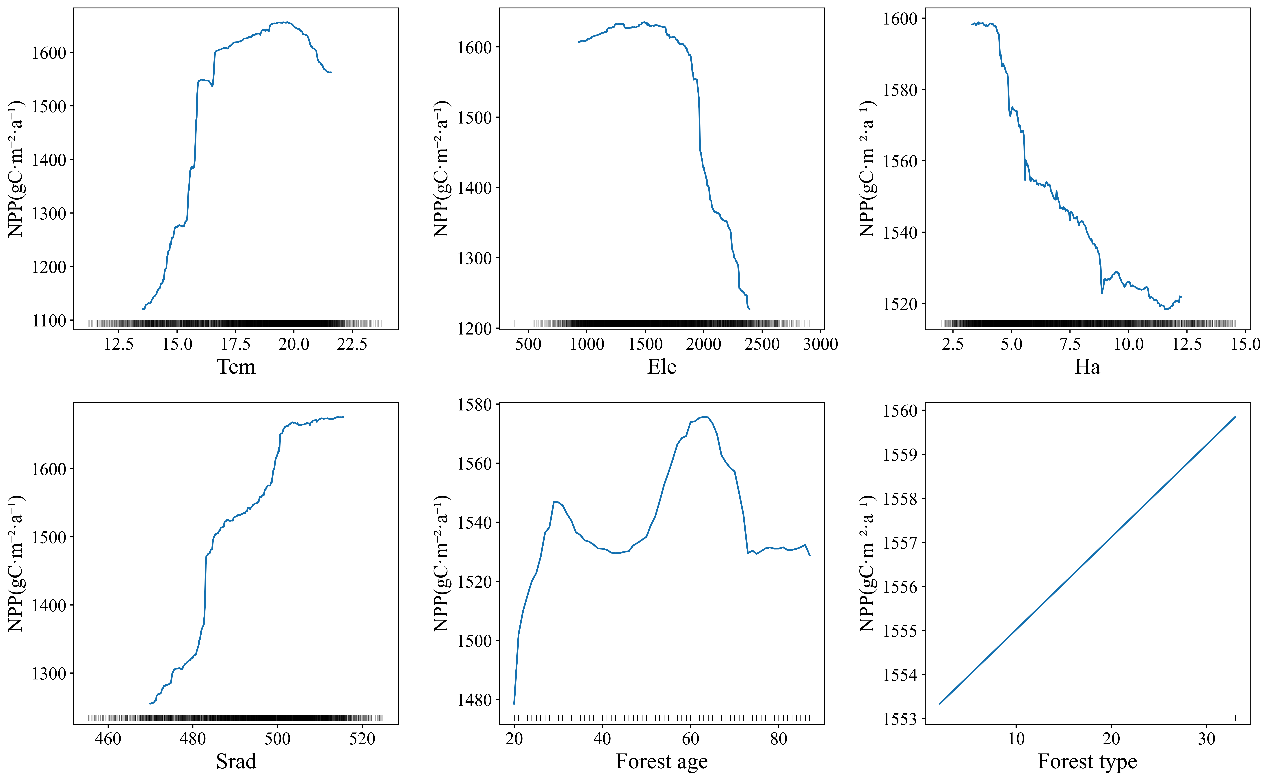
**

**Figure S11:** Partial dependence of annual mean forest NPP on driving factors in southwestern Yunnan within the stable forest.


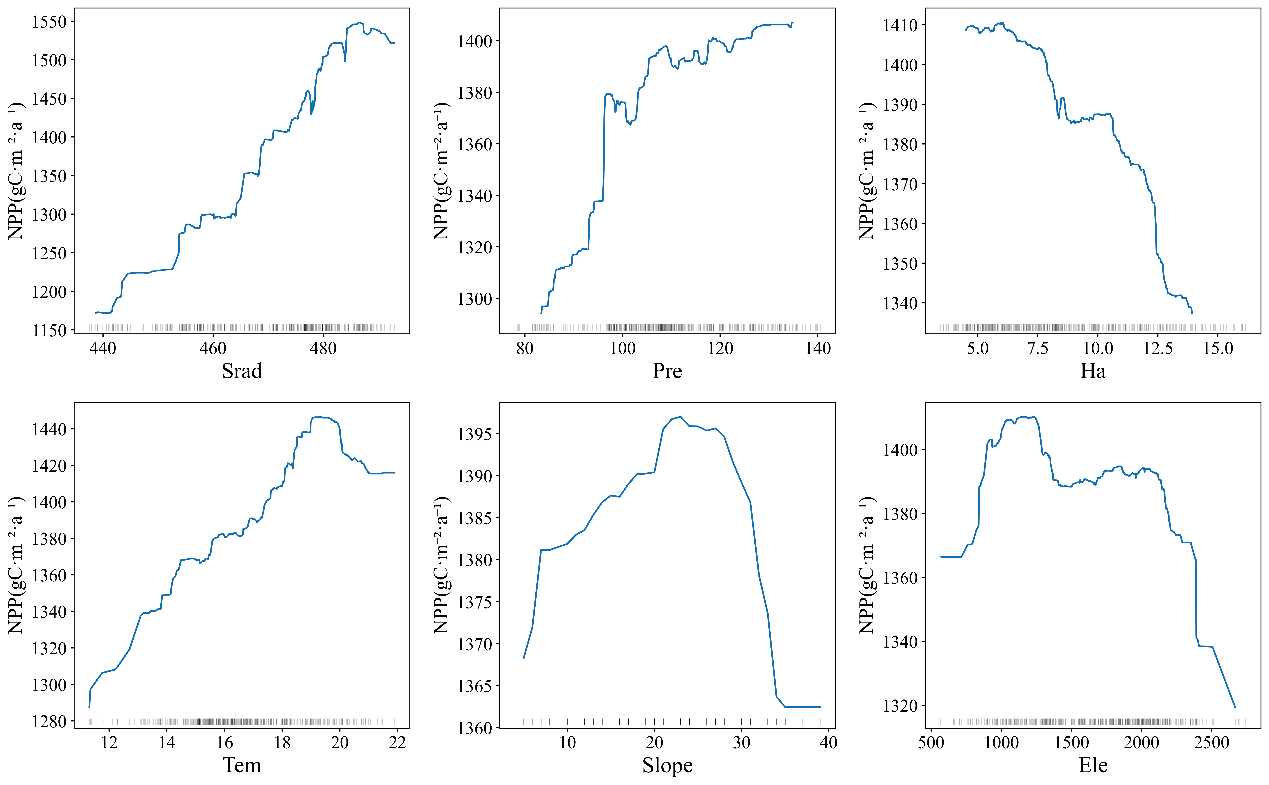


**Figure S12:** Partial dependence of annual mean forest NPP on driving factors in southeastern Yunnan within the stable forest.

**
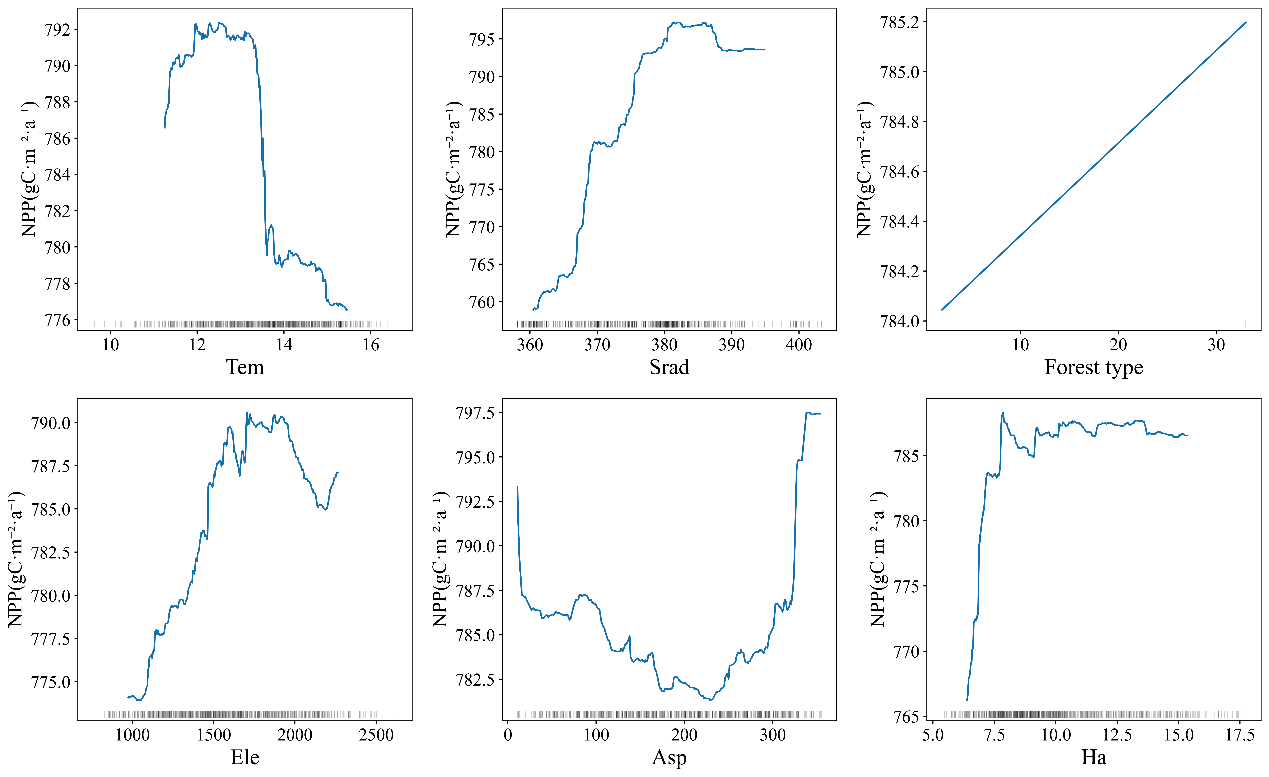
**

**Figure S13:** Partial dependence of annual mean forest NPP on driving factors in northeastern Yunnan within the changing forest.

**
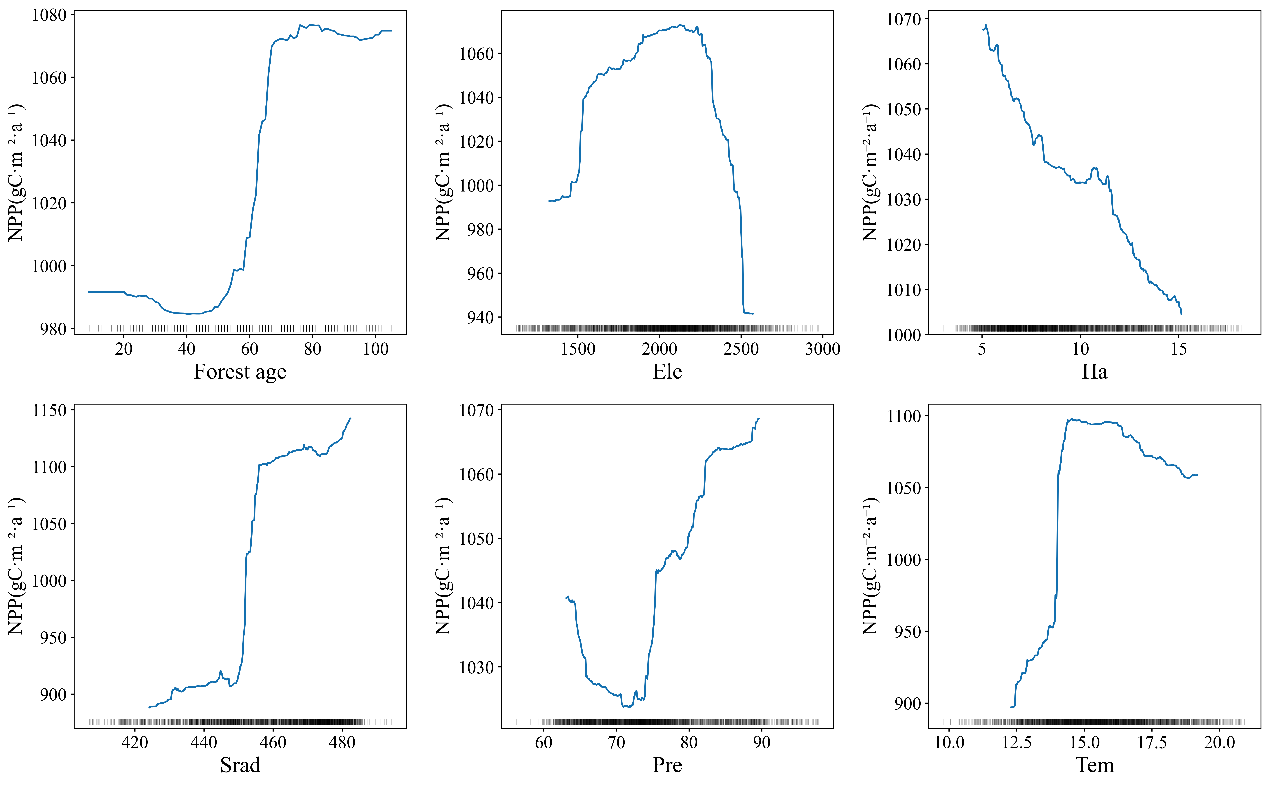
**

**Figure S14:** Partial dependence of annual mean forest NPP on driving factors in central Yunnan within the changing forest.


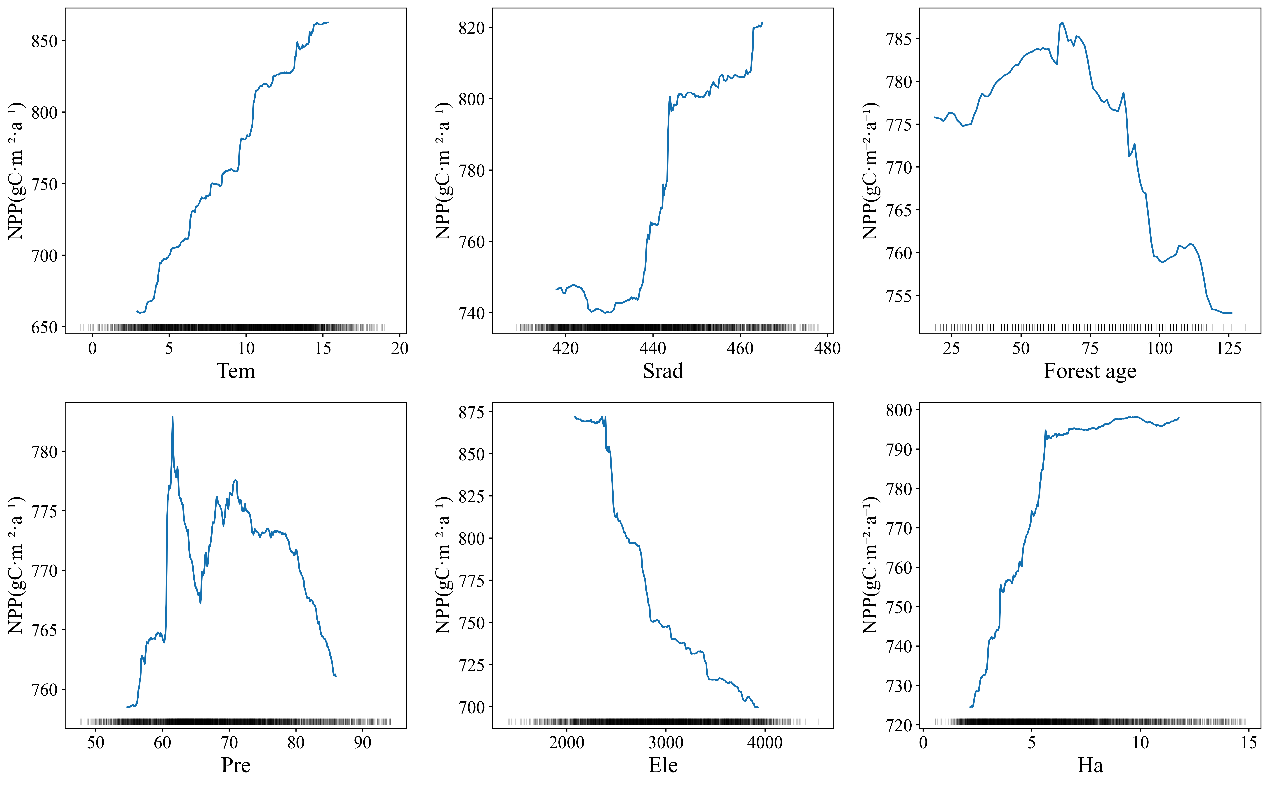


**Figure S15:** Partial dependence of annual mean forest NPP on driving factors in northwestern Yunnan within the changing forest.

**
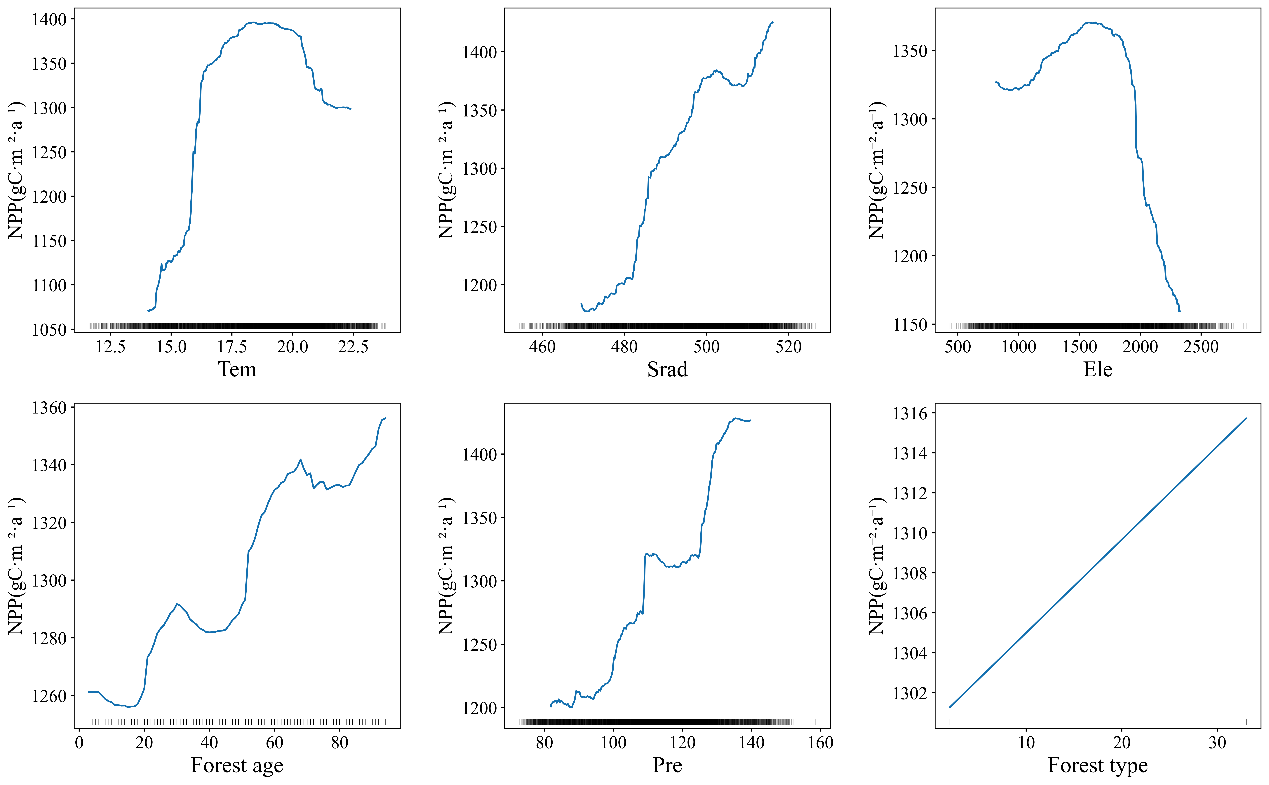
**

**Figure S16:** Partial dependence of annual mean forest NPP on driving factors in southwestern Yunnan within the changing forest.

**
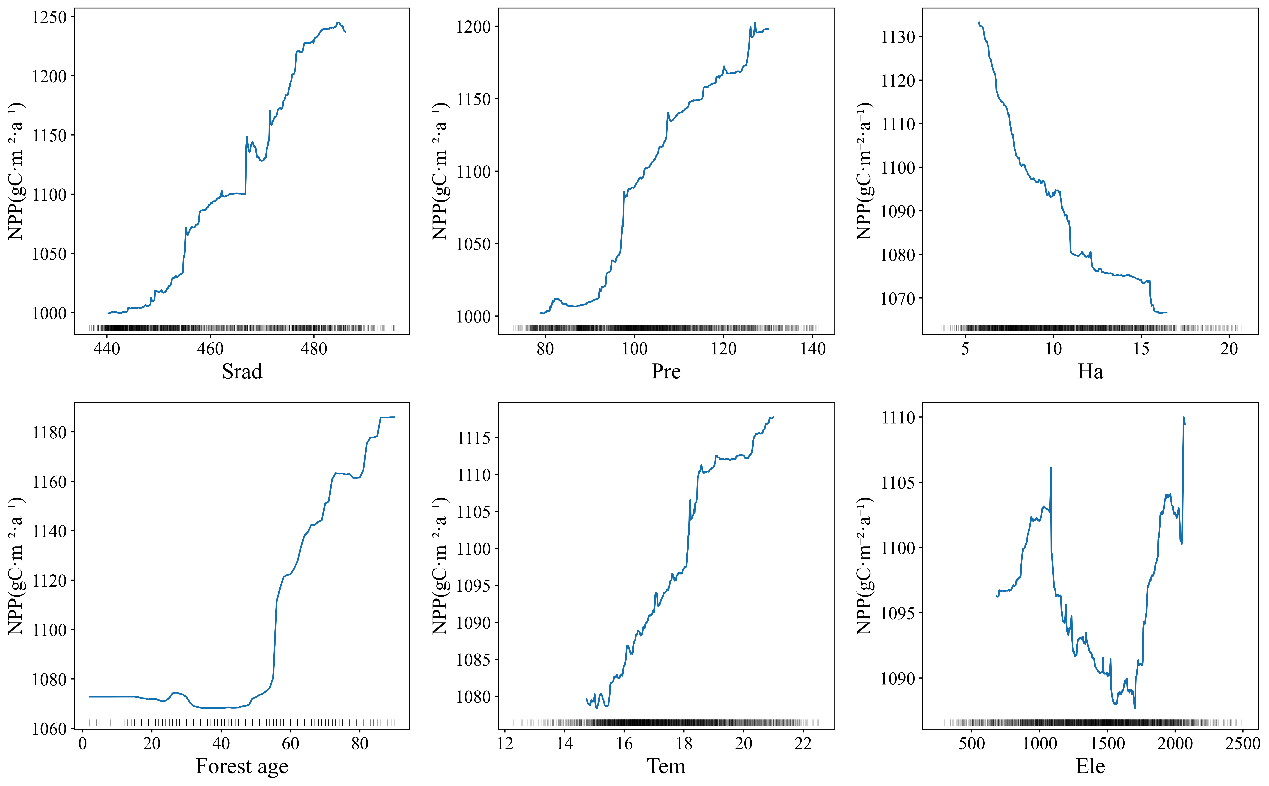
**

**Figure S17:** Partial dependence of annual mean forest NPP on driving factors in southeastern Yunnan within the changing forest.


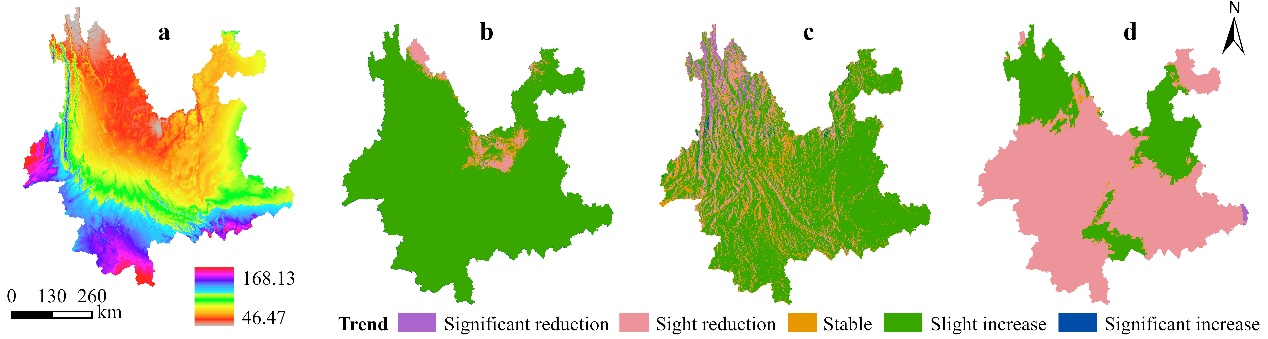


**Figure S18：** Distribution of monthly average precipitation (a), trends in precipitation (b), solar radiation (c), and temperature (d).

References

Zhu, W., Pan, Y., He, H., Yu, D., Hu, H., 2006. Simulation of maximum light use efficiency for some typical vegetation types in China. Chin. Sci. Bull. 51, 457–463.
